# Molecularly Imprinted Polymers for Chemical Sensors: From Molecular Recognition to Advanced Architectures and Practical Sensing Platforms

**DOI:** 10.3390/s26217087

**Published:** 2026-11-09

**Authors:** Marcos Vinicius Foguel, Severino Marcelino dos Santos-Neto, Suennia Carinne da Silva Sena, Flávia Suellen Melo de Oliveira, Írinan Barbosa de França, Bianca Paula Silva de Albuquerque, Victória Costa, Marcella Auxiliadora de Melo Lucena, Marcella Eduarda de Melo Ferreira, Mariana de Mendonça Martelli, Márcio Paulo do Nascimento, César Vinicius dos Santos Martins Caneja, Alice Braga de Souza

**Affiliations:** Department of Fundamental Chemistry, Federal University of Pernambuco, Recife 50740-560, PE, Brazil; severino.neto@ufpe.br (S.M.d.S.-N.); suennia.sena@ufpe.br (S.C.d.S.S.); flavia.suellen@ufpe.br (F.S.M.d.O.); irinan.franca@ufpe.br (Í.B.d.F.); bianca.paula@ufpe.br (B.P.S.d.A.); victoria.vc@ufpe.br (V.C.); marcella.lucena@ufpe.br (M.A.d.M.L.); marcella.mferreira@ufpe.br (M.E.d.M.F.); mariana.martelli@ufpe.br (M.d.M.M.); marcio.paulon@ufpe.br (M.P.d.N.); cesar.caneja@ufpe.br (C.V.d.S.M.C.); alice.bsouza@ufpe.br (A.B.d.S.)

**Keywords:** molecularly imprinted polymer, molecular recognition, chemical sensor, electrochemical sensor, electropolymerization, surface imprinting, nanoMIPs, analytical validation

## Abstract

**Highlights:**

**What are the main findings?**

- MIP-based sensors have evolved from conventional bulk materials toward surface-oriented, nanoscale, and hybrid recognition architectures.
- Recognition-site accessibility, mass transfer, and transducer integration are key determinants of MIP sensor performance.

**What is the implication of the main finding?**

- Emerging approaches such as nanoMIPs, epitope imprinting, computational design, and miniaturized platforms expand MIP applications toward complex targets and practical sensing formats.
- Analytical validation, response time, regeneration efficiency, reproducibility, scale-up, and device-level quality control are critical for translating MIP sensors into practical applications.

**Abstract:**

Molecularly imprinted polymers (MIPs) are synthetic recognition materials that combine molecular selectivity with chemical and structural stability, making them attractive for chemical sensing. This review examines the development of MIP-based sensors, from conventional imprinting strategies to advanced architectures and integrated sensing platforms, with emphasis on the relationship between material structure, molecular recognition, and signal transduction. Classical preparation methods, including bulk, precipitation, emulsion, suspension, and sol–gel polymerization, are compared in terms of morphology, recognition-site accessibility, mass transfer, and processing requirements. Advanced architectures, including magnetic MIPs, carbon-based hybrids, hollow and restricted-access systems, nanoMIPs, nanogels, and molecularly imprinted hybrid materials, are discussed in relation to their integration into electrochemical and optical transducers. Particular attention is given to electropolymerization, epitope imprinting, solid-phase nanoMIP synthesis, computationally guided MIP design, and portable, microfluidic, paper-based, and wearable sensing platforms. The effects of MIP architectures and preparation strategies on response time, regeneration requirements and efficiency, and operational stability are also examined in the context of practical sensor performance. A bibliometric analysis based on Scopus and Web of Science is presented to contextualize the growth and diversification of the field. Beyond analytical sensitivity and low detection limits, this review emphasizes the importance of validation in realistic matrices, reproducibility, stability, manufacturing control, and appropriate benchmarking. Overall, the practical development of MIP-based sensors depends on the effective integration of molecular recognition with the transducer and on reproducible control of the entire recognition–transduction interface.

## 1. Introduction

Molecularly imprinted polymers (MIPs) have emerged as synthetic recognition materials capable of mimicking natural recognition systems. They combine molecular selectivity with chemical stability and relatively low cost compared with biological receptors [1,2,3,4]. These characteristics have attracted considerable attention in analytical chemistry, particularly for the analysis of complex matrices [5,6,7,8]. Their robustness and adaptability to different chemical environments also make MIPs attractive alternatives to biological recognition elements, such as enzymes and antibodies [9,10,11].

When evaluating MIP-based sensors, it is important to distinguish specific molecular recognition from non-specific adsorption. Non-imprinted polymers (NIPs), prepared under the same experimental conditions as their corresponding MIPs but without the template molecule, are commonly used as control materials. Comparing the analytical responses of MIPs and NIPs at matched analyte concentrations and under equivalent experimental conditions helps assess the contribution of the imprinting process to analyte recognition. Such comparisons should also consider differences in polymer morphology, surface area, porosity, and recognition-site accessibility, since these properties can influence the observed responses [12].

**Figure 1:**
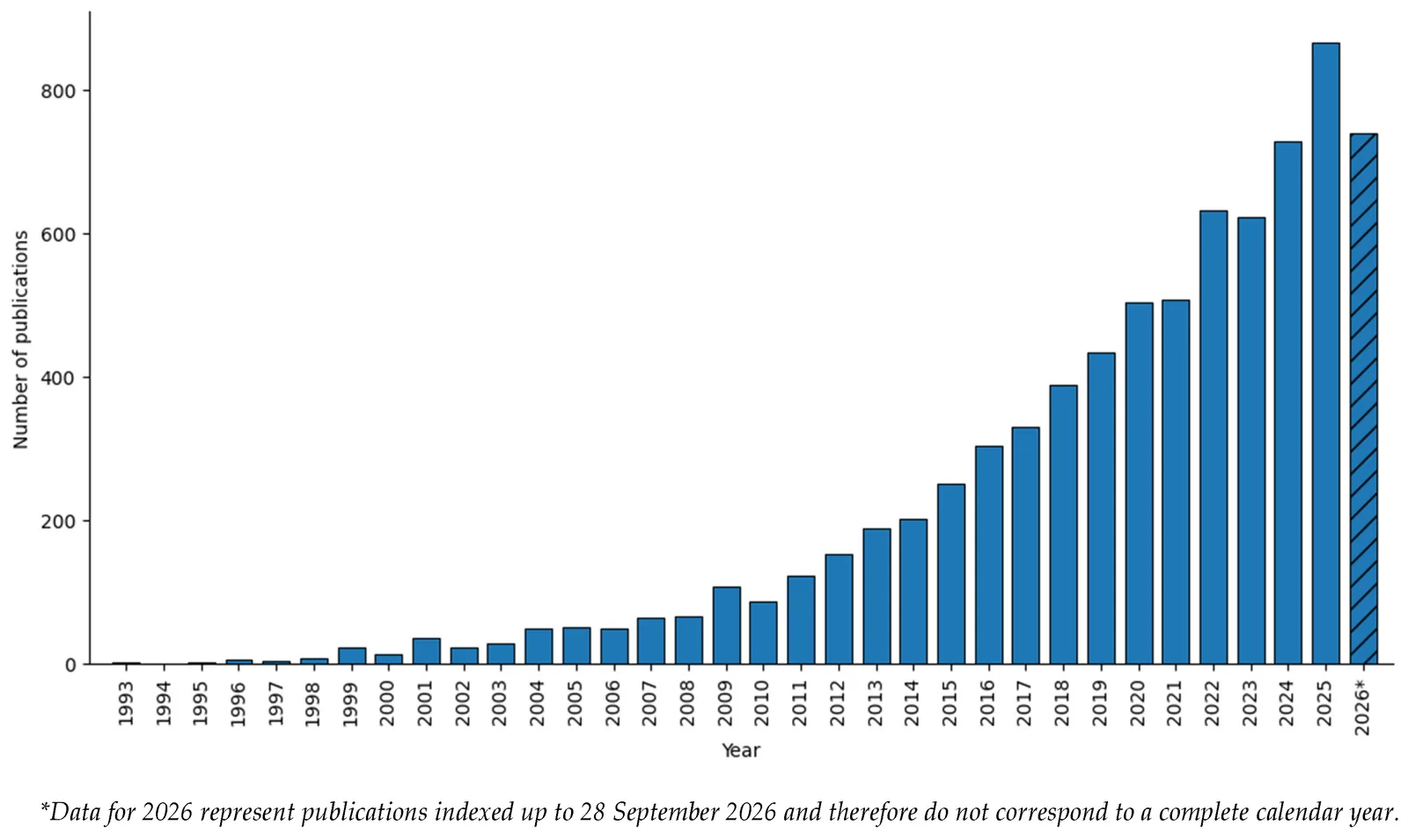
Annual scientific production related to MIP-based sensing from 1993 to 2026, based on the deduplicated corpus retrieved from Scopus and the Web of Science Core Collection using the search terms “molecularly imprinted polymer” AND “sensor*”.

Over the past few decades, the use of MIPs in sensor development has increased considerably. A bibliometric analysis combining records retrieved from the Scopus and Web of Science Core Collection databases identified 7580 unique publications related to MIP-based sensing after duplicate records were removed. Of these, 6840 were published between 1993 and 2025, and 5317 (77.7%) appeared between 2016 and 2025. Annual scientific production increased from 304 publications in 2016 to 866 in 2025, while 740 publications had already been indexed by 28 September 2026, although the year was not yet complete (Figure 1). These data illustrate the marked growth of research on MIP-based sensing, particularly over the past decade.

**Figure 2:**
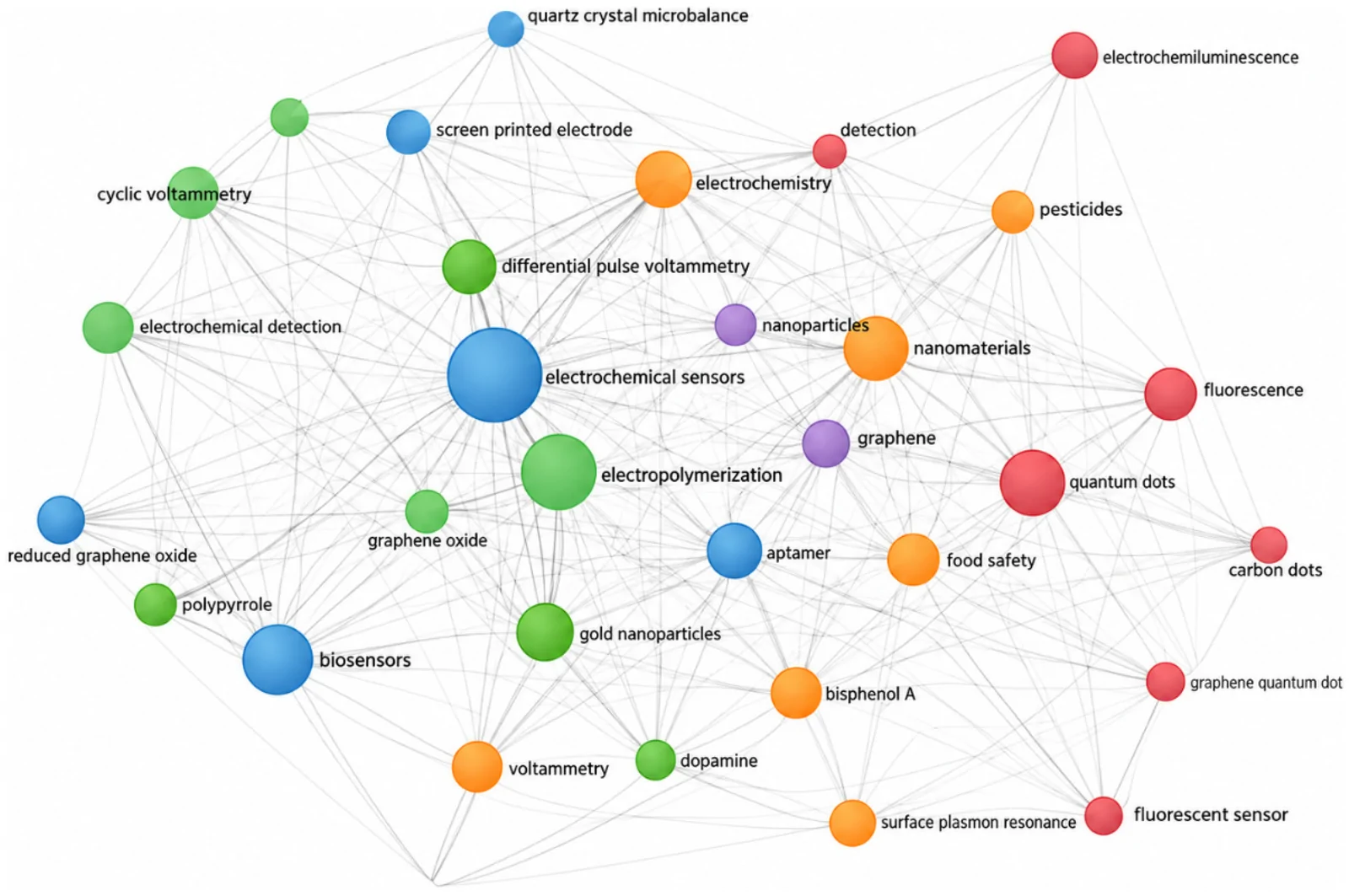
Keyword co-occurrence network of MIP-based sensing research during 2016–2025. Node size represents normalized keyword frequency, node color indicates the thematic community identified by clustering, and link width represents co-occurrence strength.

This increase has been accompanied by a diversification of research topics and sensing strategies. Keyword co-occurrence analysis for 2016–2025 identified electrochemical sensors, biosensors, electropolymerization, metal–organic frameworks, quantum dots, aptamer-based systems, food safety, differential pulse voltammetry, nanomaterials, and gold nanoparticles among the most frequent terms. Optical and mass-sensitive approaches, including fluorescence and quartz crystal microbalance, were also represented (Figure 2). The results reflect the growing range of materials and transduction strategies used in MIP-based sensing. Reported platforms include electrochemical, optical, piezoelectric, and spectroscopic systems [13,14,15,16,17,18], with fabrication approaches involving sol–gel processing, electrosynthesis, and the incorporation of nanomaterials into imprinted architectures [19,20,21]. Electrochemical platforms are widely investigated because of their compatibility with electrode modification, relatively simple instrumentation, and potential for miniaturization. Optical approaches, including fluorescence, surface-enhanced Raman scattering (SERS), and surface plasmon resonance (SPR), provide complementary means of signal detection [22,23]. Although both approaches are considered, electrochemical platforms are more extensively represented in this review, reflecting the larger number of studies reported in the literature.

**Figure 3:**
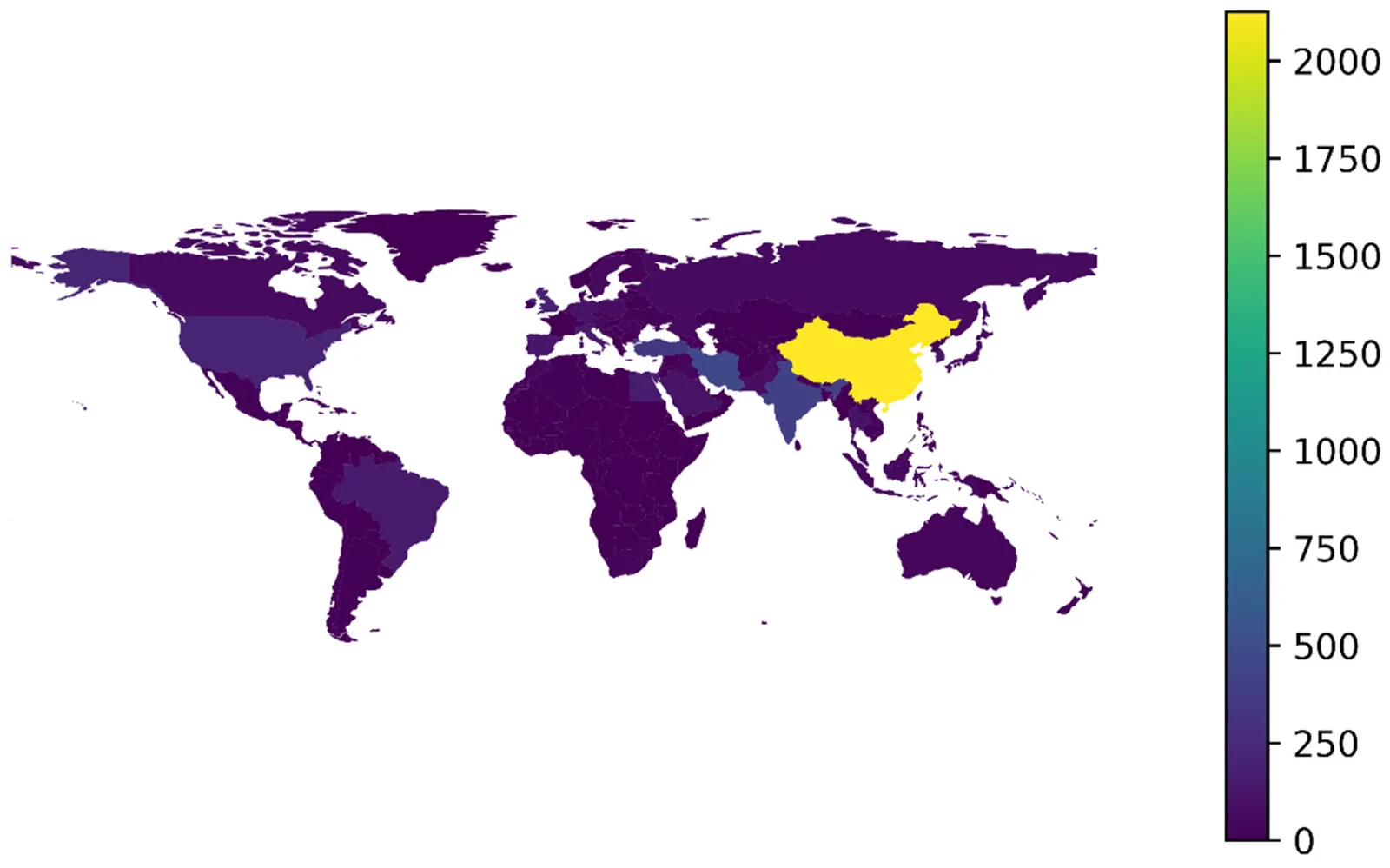
The geographical distribution of publications on MIP-based sensing during 2016–2025 based on the deduplicated Scopus and Web of Science corpus. Countries are represented according to the number of publications associated with author affiliations using whole counting.

The geographical distribution of publications also reflects the international development of MIP-based sensing. During 2016–2025, scientific production was concentrated in a limited number of countries, particularly China, Iran, India, and Türkiye. Relevant contributions were also identified from the United States, the United Kingdom, Italy, Brazil, and other countries across Asia, Europe, the Americas, and the Middle East (Figure 3).

Despite this growth and diversification, the literature remains fragmented, with studies often focusing on individual synthesis methods, functional materials, target analytes, or transduction mechanisms [24,25,26]. This makes direct comparisons between sensing systems difficult, since polymer morphology, recognition-site accessibility, mass transfer, surface organization, and integration with the transducer can all affect analytical performance, even when similar imprinting chemistries are used [27]. Improved analytical figures of merit under controlled laboratory conditions do not necessarily translate into adequate reproducibility, stability, scalability, or practical applicability [18,28]. Response time and regeneration are also relevant operational parameters. The time required to reach a measurable response depends, among other factors, on analyte diffusion through the polymer and access to the recognition sites [29]. Similarly, regeneration depends on the ability to remove the bound analyte without compromising the polymer structure or the sensing interface [8,30]. These aspects should therefore be considered alongside sensitivity and detection limits when assessing the practical performance of MIP-based sensors [30,31].

This review examines the different stages involved in MIP-based sensor development. It first discusses the fundamental principles of molecular imprinting and compares conventional preparation strategies in terms of their effects on morphology, recognition-site accessibility, mass transfer, and processing requirements. The main advanced architectures and functional modifications developed to address the limitations of conventional MIPs are then examined, including magnetic, carbon-based, hollow, restricted-access, nanoscale, and hybrid systems. The review also examines the integration of MIPs with electrochemical and optical transducers, the influence of material organization on molecular recognition and signal transduction, and current challenges in analytical validation, biomacromolecular recognition, computationally guided imprinting, sustainable and scalable synthesis, portable and miniaturized platforms, reproducibility, and manufacturing. The aim is to relate developments in MIP materials and architectures to the requirements of practical chemical sensing.

To support this analysis, a bibliometric survey was conducted using the Scopus and Web of Science Core Collection databases. Equivalent search concepts were applied using TITLE-ABS-KEY(“molecularly imprinted polymer*” AND sensor*) in Scopus and TS=(“molecularly imprinted polymer*” AND sensor*) in Web of Science. Records classified as Article or Review and published between 1993 and 2026 were considered. Duplicate records were removed primarily using DOI matching and, when DOI information was unavailable, by normalized exact-title matching, resulting in a final deduplicated corpus of 7580 records. Annual scientific production was evaluated from 1993 to 2026, with 2026 retained as a partial-year snapshot because the data were retrieved on 28 September 2026. For the analysis of current thematic and geographical patterns, only the complete period from 2016 to 2025 was considered. Author keywords were harmonized before co-occurrence analysis to merge clear spelling, singular/plural, and abbreviation variants, whereas generic terms directly corresponding to the search strategy were excluded from the network visualization. Geographical distribution was determined from author affiliations using whole counting. The retrieved data were analyzed using the Bibliometrix package in R and its graphical interface, Biblioshiny [32].

## 2. Fundamentals of Molecular Imprinting

The concept of molecular imprinting is based on the formation of selective recognition sites within a synthetic polymer through interactions established between a template molecule and functional monomers. The underlying principle is related to early models of molecular recognition, particularly Fischer’s enzyme–substrate “lock-and-key” model and studies of antigen–antibody interactions [33,34]. In these systems, molecular recognition results from the complementarity between the target and the recognition site. Molecular imprinting applies a similar concept to synthetic materials by using the target molecule, or a structural analog of it, as a template during the formation of the polymer network.

The first experimental approaches to molecular imprinting date back to the 1930s, when Polyakov reported selective adsorption in silica gels prepared in the presence of organic molecules. In 1949, Dickey further demonstrated that silica-based materials could exhibit preferential affinity toward specific compounds, providing early evidence that the organization of a material in the presence of a target molecule could influence its subsequent adsorption behavior [35]. Although these early systems were based on inorganic materials, they established an important principle for molecular imprinting: the properties of the resulting material depend not only on its chemical composition, but also on the conditions under which the recognition sites are formed.

A major development occurred in the 1970s with the application of molecular imprinting to cross-linked organic polymers. Wulff and Sarhan demonstrated covalent imprinting strategies in which the template was incorporated into the polymer network through reversible covalent interactions, establishing a more controlled approach for the formation of binding sites [36]. Subsequent studies by Shea and Mosbach introduced and further developed non-covalent imprinting strategies based on reversible interactions between the template and functional monomers [37,38]. In contrast to covalent imprinting, non-covalent interactions do not require the formation and subsequent cleavage of specific covalent bonds. This characteristic provides greater flexibility in the choice of templates and functional monomers and facilitates template removal and rebinding. As a result, non-covalent imprinting became the most widely investigated approach for the preparation of MIPs [39].

In a typical molecular imprinting process, the template molecule is first allowed to interact with one or more functional monomers. The interactions established at this stage determine, to a large extent, the chemical environment of the future recognition sites. Polymerization is then performed in the presence of a crosslinking agent and an initiator, generating a rigid polymer network around the template–monomer complexes. After polymerization, the template is removed from the polymer, leaving cavities that are complementary to the target molecule in terms of size, shape, and distribution of functional groups. The resulting sites can selectively rebind the template or structurally related molecules. Therefore, the recognition properties of an MIP depend on both the chemical interactions responsible for template–monomer association and the physical structure of the polymer network [40,41].

NIPs are useful reference materials for investigating the recognition properties of MIPs. Although prepared without the template molecule, NIPs may still exhibit adsorption sites arising from the polymer composition, morphology, and porosity. Their use therefore helps distinguish the contribution of the imprinted recognition sites from adsorption associated with the polymer matrix itself [42,43,44].

The interactions established during imprinting depend on the chemical characteristics of the template and functional monomers and are commonly used to classify MIPs as covalent, non-covalent, semi-covalent, or metal-mediated systems [45]. In covalent imprinting, the template forms reversible covalent bonds with the functional monomer before or during polymer formation. This strategy can provide well-defined binding sites and controlled template–monomer stoichiometry. However, the chemical groups involved must be compatible with reversible bond formation, and the subsequent cleavage of these bonds may complicate template removal and rebinding [36,46]. These requirements can restrict the range of molecules that can be successfully imprinted using a covalent approach.

Non-covalent imprinting is based on reversible interactions such as hydrogen bonding, electrostatic interactions, van der Waals forces, and hydrophobic interactions. Because these interactions do not require chemical modification of the template, a broad range of molecules can be investigated, and template removal is generally simpler than in covalent systems [47,48]. However, the same weak and reversible nature of these interactions can also affect the organization of the recognition sites. Differences in the strength and number of interactions established between the template and functional monomers can result in binding sites with different affinities, contributing to heterogeneous binding behavior. Thus, although non-covalent imprinting offers greater synthetic flexibility, the control of the interactions established during polymer formation remains an important factor in determining the final recognition properties of the MIP.

Semi-covalent imprinting combines covalent interactions during polymer formation with non-covalent interactions during the subsequent recognition step [47,48,49]. In this approach, covalent interaction can be used to control the organization of the template during polymerization, whereas rebinding occurs through weaker non-covalent interactions after removal of the template. Metal-mediated imprinting provides another possibility for controlling template–monomer interactions. In these systems, metal ions participate in coordination interactions involving the template and functional monomers, generating recognition sites in which the coordination environment contributes to target selectivity [38,45,46]. This approach has been explored, for example, in ion-selective electrodes and remediation applications.

The choice of imprinting strategy directly affects the interactions established during polymer formation and, consequently, the structure and recognition properties of the resulting MIP. However, the imprinting mechanism is only one of the parameters determining the final performance of the material. The template-to-monomer ratio, type and concentration of functional monomers, crosslinking density, solvent, initiator, polymerization conditions, and template removal procedure can all influence the formation and accessibility of the recognition sites [50]. These parameters can affect binding capacity, selectivity, mass transfer, and reproducibility. For sensor applications, these effects become particularly important because recognition occurs at or near a transducer surface, where the accessibility of the binding sites and the transport of the analyte to these sites can determine whether the molecular recognition event is effectively converted into an analytical signal.

## 3. MIP Preparation Methods

The properties of an MIP depend not only on the chemical interactions established between the template and functional monomers, but also on the method used to form the polymer network. The polymerization strategy affects the morphology, particle size, and porosity of the material, the distribution and accessibility of the recognition sites, and analyte transport through the polymer matrix. These characteristics are particularly relevant for sensing applications because the recognition sites must remain accessible to the analyte and the resulting binding event must occur under conditions compatible with the transducer [50,51].

**Figure 4:**
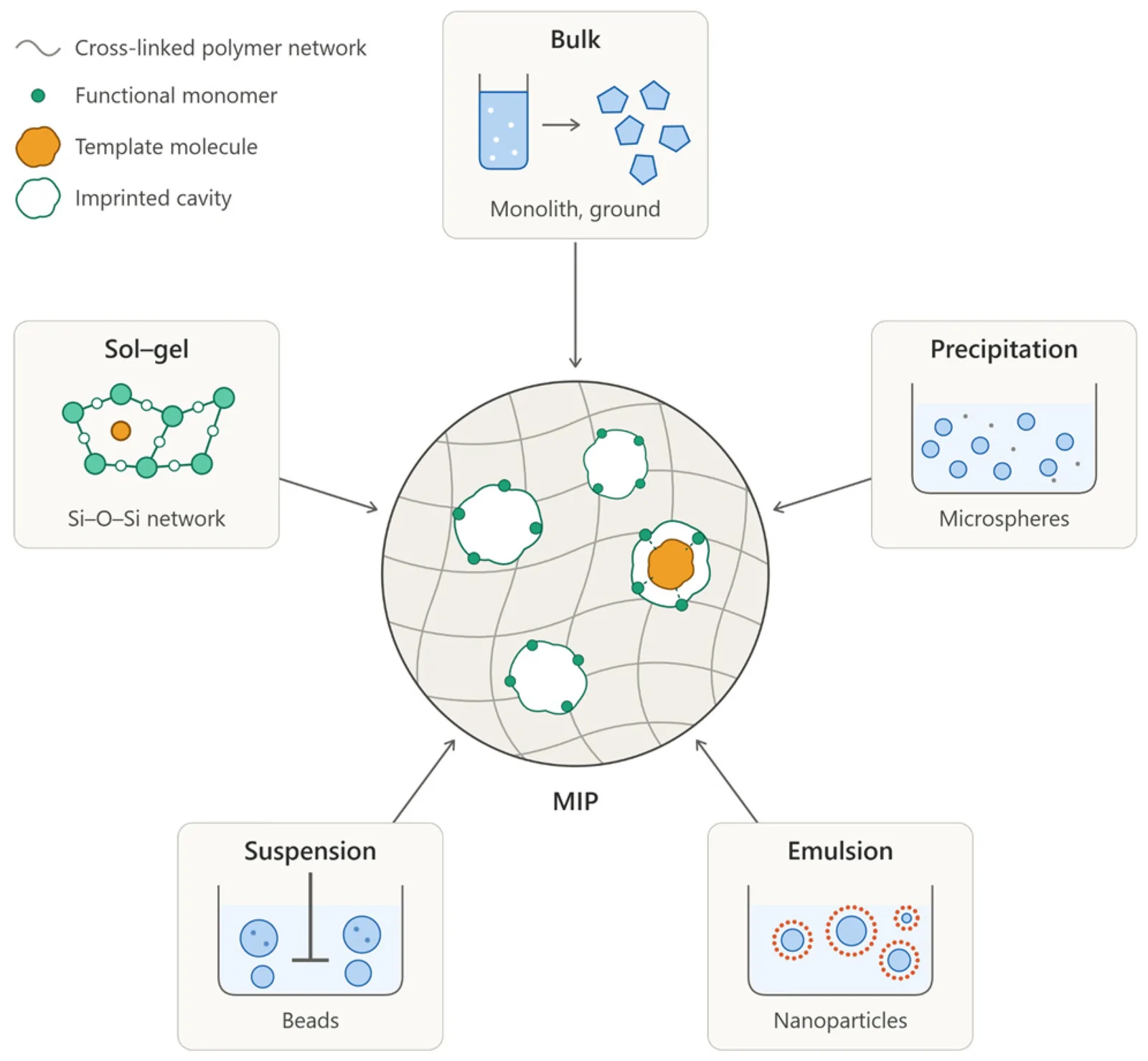
A schematic representation of the main methods used for MIP preparation, including bulk, precipitation, suspension, emulsion, and sol–gel polymerization, illustrating the different synthetic approaches and the resulting imprinted polymer network.

Several approaches have been developed for MIP preparation, including bulk, precipitation, emulsion, suspension, and sol–gel polymerization. In addition, polymerization can be performed directly on a substrate or transducer surface, allowing the recognition material to be integrated with the sensing interface [5,46]. Although these approaches are based on the same general principle of forming a polymer in the presence of a template, the resulting materials can differ substantially in particle size, morphology, porosity, thickness, and recognition-site accessibility. Therefore, the choice of polymerization method is not only a synthetic consideration but can also determine how the MIP behaves when incorporated into a sensor [52]. A schematic overview of the main MIP preparation methods and their representative resulting architectures is presented in Figure 4.

### 3.1. Bulk Polymerization

Bulk polymerization is one of the most traditional approaches for preparing MIPs because of its simple procedure, low cost, and versatility [53,54]. In this method, the template, functional monomer, crosslinker, and initiator are polymerized in a homogeneous reaction mixture, generally in the presence of a porogenic solvent, resulting in a highly crosslinked monolithic material [55].

Despite its simplicity, bulk polymerization has limitations related mainly to the processing of the polymer after synthesis. The resulting monolith generally needs to be mechanically ground and sieved to obtain particles of a suitable size for subsequent applications. This process produces particles with irregular shapes and a relatively broad size distribution and can expose or damage recognition sites during grinding [56]. In addition, differences in particle size and morphology can result in differences in the accessibility of the binding sites and, consequently, in the mass transfer and rebinding kinetics of the analyte. These characteristics can affect the reproducibility of molecular recognition and the analytical response, particularly when the MIP is incorporated into a sensing interface [57].

Nevertheless, bulk polymerization remains widely used for the preparation of MIPs because the method requires relatively simple experimental procedures and can be applied to different templates and functional monomers [58]. Its main advantage is therefore related to the simplicity of polymer preparation, whereas control over particle morphology and recognition-site accessibility is more limited than in methods specifically developed for particle size or surface control.

Table 1 presents representative examples of MIP-based sensors prepared by bulk polymerization, covering different analytes, synthesis components, sample matrices, and transduction methods. All seven examples employ electrochemical transduction (potentiometric, voltammetric, or capacitive), reflecting the ease with which MIPs can be integrated onto electrode surfaces and their recognition events converted into measurable electrical signals.

Regarding the performance parameters reported for MIP-based sensors prepared by bulk polymerization, the potentiometric sensors exhibited response times of up to 30 s, indicating relatively fast responses. Broad linear ranges were also achieved, as demonstrated by the potentiometric sensor developed for daclatasvir determination, which exhibited a linear range from 0.01 to 10,000 µM. Regarding the limits of detection (LOD), the voltammetric sensors developed for chlorpyrifos and metribuzin determination are noteworthy, with values of 0.01912 µM and 0.0005 µM, respectively. The reported sensitivity values also varied among the sensors, reflecting differences in the transduction mechanisms and sensor configurations.

### 3.2. Precipitation Polymerization

Precipitation polymerization is widely used for MIP synthesis because it can produce relatively uniform, spherical particles without the grinding and sieving steps required after bulk polymerization. In this approach, the template, functional monomer, crosslinker, and initiator are initially dissolved in a suitable solvent. As polymerization proceeds, the growing polymer chains become insoluble in the reaction medium, resulting in phase separation and the formation of polymer particles [66,67].

Compared with bulk polymerization, precipitation polymerization favors the formation of spherical particles with relatively narrow size distributions. The resulting morphology can increase the accessibility of surface and near-surface recognition sites and facilitate analyte transport to these sites [68,69]. These characteristics can contribute to faster binding kinetics and, depending on the polymer composition and porosity, increase the fraction of binding sites that is effectively accessible to the analyte. This feature is particularly relevant for sensing applications, in which rapid interaction between the analyte and the recognition sites is required to obtain a measurable analytical response.

The particle size and morphology obtained by precipitation polymerization depend on several experimental parameters, including solvent composition, monomer concentration, crosslinker content, template concentration, and polymerization conditions. Changes in these parameters can affect the nucleation and growth of the polymer particles and, consequently, their final size and morphology. Therefore, optimization of the synthesis conditions is required to obtain reproducible particle characteristics. In addition, the method commonly requires relatively large volumes of solvent, which can increase solvent consumption, processing costs, and environmental burden [70,71,72].

Thus, precipitation polymerization provides an alternative to bulk polymerization when greater control over particle morphology and size is required. Its main advantage for sensing applications is the formation of relatively uniform particles without the mechanical processing required for bulk polymers, which can favor the accessibility of recognition sites. However, the dependence of particle formation on the reaction conditions and the relatively high solvent consumption remain important limitations [73].

Table 2 summarizes representative MIP-based sensors prepared by precipitation polymerization. The analytical parameters presented highlight the diversity of performance achieved by MIP-based sensors prepared through precipitation polymerization. The reported response times range from 10 s to 10 min, while the linear concentration ranges vary considerably among the investigated systems. Notably, the lowest LOD reported was 0.2 µM for p-nitrophenol detection, indicating the ability of this sensor to detect relatively low analyte concentrations.

These variations reflect the influence of different synthesis compositions, analyte characteristics, and sensing configurations on the analytical performance of the resulting materials. Therefore, the reported data emphasizes the versatility of precipitation polymerization for developing MIP-based sensing systems with different analytical capabilities.

### 3.3. Emulsion Polymerization

Emulsion polymerization is an alternative strategy for MIP synthesis that can produce relatively uniform particles with controlled particle size and high surface-to-volume ratios. In this approach, polymerization occurs in a continuous phase, typically an aqueous medium, in the presence of surfactants that promote the formation and stabilization of the polymer particles [77].

The particle morphology obtained by emulsion polymerization can increase the accessibility of surface recognition sites and facilitate analyte transport, which is particularly relevant for sensing and separation applications. In addition, the use of an aqueous continuous phase can reduce the amount of organic solvent required during polymerization, depending on the composition of the reaction system [78,79].

However, the presence of surfactants can introduce additional steps in the preparation of the MIP. Surfactant molecules may remain associated with the polymer particles after polymerization and therefore require additional purification. Their presence can also modify the surface properties of the particles and affect interactions between the template and the polymer. For this reason, surfactant-free emulsion polymerization has been investigated as an alternative approach in which colloidal stability is achieved through other mechanisms [80,81]. This strategy can facilitate the preparation of polymer particles with cleaner surfaces and has been applied to the synthesis of imprinted nanoparticles for selective recognition [80,81].

### 3.4. Suspension Polymerization

Suspension polymerization is a heterogeneous technique in which the monomer mixture is dispersed as droplets in a continuous phase, typically water, in the presence of stabilizing agents. Polymerization occurs within the dispersed droplets, resulting in spherical polymer particles with relatively uniform sizes [82].

Compared with bulk polymerization, suspension polymerization eliminates the need for grinding and sieving of the polymer after synthesis and provides greater control over particle size and morphology [83]. The formation of spherical particles can also improve the accessibility of recognition sites and facilitate analyte transport compared with the irregular particles obtained after mechanical processing of bulk polymers.

The particle size and stability of the suspension depend strongly on experimental parameters such as agitation rate, stabilizer concentration, phase composition, and monomer concentration. These parameters affect the formation and stability of the droplets and must therefore be controlled to prevent droplet coalescence and particle aggregation and to obtain reproducible particle characteristics [82]. In addition, residual stabilizers may require additional purification and can modify the surface properties of the final MIP.

Suspension polymerization has been applied to the preparation of MIPs for different analytical applications, including electrochemical sensing [84,85].

### 3.5. Sol–Gel Techniques

Sol–gel chemistry provides an alternative route for preparing molecularly imprinted inorganic and hybrid materials, particularly for optical and electrochemical sensing applications. The process is based on the hydrolysis and condensation of suitable precursors, commonly silica-based compounds, in the presence of the template, resulting in an inorganic or hybrid network containing molecular recognition sites [86,87,88].

One of the main advantages of sol–gel synthesis is the possibility of controlling pore structure, surface area, and chemical functionality through the choice of precursors and reaction conditions. These characteristics can affect analyte diffusion and the accessibility of recognition sites within the material [89,90]. The synthesis can also be performed under relatively mild conditions. However, the structure and recognition properties of the resulting material depend strongly on parameters such as pH, precursor concentration, water content, and catalyst type. The conditions used for hydrolysis and condensation can affect template–precursor interactions and, consequently, the formation and accessibility of the recognition sites [91,92].

Sol–gel-derived MIPs have been applied in different sensing platforms, including optical, piezoelectric, and electrochemical systems. In these applications, the porous inorganic or hybrid network can provide both accessible recognition sites and structural stability during sensor operation [93].

Beyond their effects on morphology, recognition-site accessibility, and mass transfer, MIP preparation strategies can also influence the regeneration requirements of the resulting sensing materials. In bulk MIPs, analyte molecules bound to recognition sites located within the polymer matrix may be more difficult to remove because of longer diffusion pathways and restricted accessibility, potentially requiring longer washing periods or more intensive elution protocols [56,57]. In contrast, architectures with recognition sites located closer to the material surface may facilitate analyte desorption by reducing diffusion distances and increasing the fraction of accessible binding sites [94,95]. However, improved accessibility does not necessarily guarantee more efficient regeneration, since desorption also depends on the strength of template–polymer interactions, polymer composition, and the chemical compatibility of the elution conditions with the recognition material [8,30]. Therefore, the selection of an MIP preparation strategy should consider not only initial analytical performance but also how polymer architecture affects analyte removal and the feasibility of regeneration under the intended operating conditions.

The limitations associated with conventional MIP preparation methods have motivated the development of alternative architectures aimed at improving binding-site accessibility, mass transfer, and integration with sensing interfaces.

## 4. Advanced MIP Architectures and Functional Modifications

Conventional MIP preparation methods can produce materials in which a substantial fraction of the recognition sites is located within the bulk polymer matrix [96]. In bulk polymerization, the grinding and sieving steps required to process the polymer can also produce irregular particles, resulting in heterogeneous recognition-site accessibility and potentially limiting analyte transport and binding kinetics [46,97,98]. These limitations have motivated the development of architectures in which the recognition sites are positioned closer to the material surface or the MIP is combined with functional supporting materials.

Surface-oriented architectures, including surface-imprinted and core–shell systems, confine the imprinted layer to or near the outer region of the material. This configuration can increase the accessibility of recognition sites, shorten the diffusion path of the analyte, and facilitate template removal compared with conventional bulk MIPs [99,100]. These characteristics are particularly relevant for sensing applications in which rapid interaction between the analyte and the recognition sites is required.

Different supporting materials have been incorporated into these architectures, including silica nanoparticles, carbon-based nanomaterials, metallic nanoparticles, and porous structures [101,102,103,104,105,106,107]. Depending on their composition and integration with the MIP, these materials can contribute to the sensing interface by increasing the accessible surface area, facilitating electron transfer, providing magnetic separation, or introducing additional optical or structural properties [99].

The development of advanced MIP architectures therefore involves not only changes in polymer composition but also control over the location and accessibility of the recognition sites and their integration with the sensing interface. The following sections discuss established support-based systems, emerging MIP architectures, and hybrid systems in which additional materials contribute to analyte transport or signal transduction.

### 4.1. Established Support-Based Architectures

#### 4.1.1. Magnetic MIPs

Magnetically responsive molecularly imprinted polymers (magnetic MIPs) combine the molecular recognition properties of MIPs with the magnetic response of magnetic nanoparticles, allowing the material to be manipulated and separated using an external magnetic field [101]. Depending on the architecture, the magnetic component can be incorporated into the polymer matrix or used as a core or support for an imprinted layer. Core–shell configurations are particularly attractive because they combine magnetic manipulation with surface-accessible recognition sites. In these systems, the magnetic core provides the magnetic response, while an intermediate protective or functional layer can provide a suitable surface for the formation of the imprinted layer [97,108].

One of the main advantages of magnetic MIPs is the possibility of rapidly recovering the material from complex matrices using an external magnetic field, reducing or eliminating the need for conventional separation steps such as centrifugation or filtration. In core–shell systems, localization of the recognition sites near the particle surface can additionally facilitate analyte transport and improve access to the binding sites, which can contribute to faster binding kinetics [109,110,111]. Thus, the magnetic component can be used not only to facilitate material recovery but also to simplify sample preparation and handling.

Magnetite (Fe_3_O_4_) is one of the most commonly used magnetic materials in these systems. However, uncoated Fe_3_O_4_ nanoparticles can undergo oxidation and aggregation, which can affect their magnetic and colloidal properties. Silica or polymer coatings are therefore frequently introduced to improve particle stability and, in the case of silica, to provide functional groups for the subsequent formation of the MIP layer [103,104]. In Fe_3_O_4_@SiO_2_-based architectures, surface silanol groups can be further functionalized with polymerizable silanes such as 3-(methacryloxy)propyltrimethoxysilane (MPS), providing anchoring sites for the formation of the imprinted layer [112,113].

The role of the magnetic component depends on how the material is incorporated into the analytical system. In sample preparation, magnetic MIPs can be used for selective extraction or preconcentration followed by rapid magnetic recovery. In integrated sensing systems, the magnetic response can also be used to concentrate analyte-loaded MIPs at the transducer surface, facilitating the assembly and positioning of the recognition material at the sensing interface. When the magnetic support also exhibits conductive, catalytic, or optical properties, it can contribute directly to signal generation rather than functioning only as a means of material separation [97].

However, increasing the magnetic content can modify the morphology and surface chemistry of the particles and reduce the relative amount of imprinted polymer available for molecular recognition [108]. A higher magnetic fraction may improve magnetic responsiveness but, depending on the architecture, decrease the number or accessibility of recognition sites. The composition and distribution of the magnetic and polymeric phases must therefore be controlled to obtain an adequate balance between magnetic manipulation and recognition performance.

Magnetic MIPs have been investigated for environmental, food, and biomedical applications, particularly when magnetic manipulation can simplify sample preparation, material recovery, or integration with sensing interfaces. Representative examples are summarized in Table 3, illustrating the different magnetic architectures, transduction methods, and target analytes reported in the literature.

#### 4.1.2. Silica Nanoparticles

Silica nanoparticles are widely used as supports for molecular imprinting because of their high specific surface area, chemical stability, and readily functionalizable surface. The presence of surface silanol groups allows for controlled functionalization and provides sites for anchoring or growing the imprinted layer [126]. In this configuration, silica acts as a structural support for the MIP and can facilitate the formation of recognition sites that are accessible at or near the particle surface.

In sensing applications, silica-supported MIPs have been investigated in potentiometric, optical, and mass-sensitive platforms, in which surface coverage, film morphology, and interfacial stability can affect the analytical response [127,128]. The porous structure and functionalizable surface of silica can also facilitate analyte transport and provide a stable substrate for the formation of the recognition layer.

Representative studies illustrate the application of these architectures in different sensing systems. A potentiometric MIP–silica sensor developed for sarcosine showed a response time of approximately 30 s and remained stable for at least 150 days [128]. In a QCM-based sensor for hexanal in grass carp filet samples, the MIP–silica system showed a strong correlation with SPME-GC-MS measurements (r = 0.98), demonstrating a close relationship between the sensor response and the reference analytical method [128].

The performance of silica–MIP systems depends on parameters such as particle size, porosity, surface functionalization, and the thickness and uniformity of the imprinted layer. These parameters affect the accessibility of the recognition sites and the interaction between the MIP and the transducer surface. Therefore, control of the silica/MIP interface is an important aspect of the design of these sensing systems [129].

#### 4.1.3. Carbon Nanotubes

Carbon nanotubes, particularly multi-walled carbon nanotubes (MWCNTs), have been widely incorporated into MIP-based electrochemical sensors because of their electrical conductivity, high specific surface area, and chemical stability [130,131,132]. Their role is not limited to increasing the available surface area. Depending on the integration strategy, MWCNTs can provide a conductive scaffold for the imprinted material or serve as a substrate for direct formation of the MIP layer. In both configurations, the nanotubes can facilitate electron transfer and increase the effective interfacial area, while the MIP provides molecular recognition [133].

Two main integration approaches are illustrated in Table 4. In surface architectures, the MIP is polymerized directly on functionalized MWCNTs, placing the recognition layer at the conductive support. Alternatively, pre-synthesized MIPs can be physically mixed with MWCNTs, providing a simpler fabrication route but potentially resulting in less controlled organization of the recognition material at the electrode interface. Surface functionalization of MWCNTs, for example with carboxyl or vinyl groups, can improve their dispersion and provide reactive sites for interaction with the polymer matrix or covalent attachment of the growing polymer [46,134].

Representative studies illustrate these different integration strategies. A suspension-polymerized MIP formed directly on vinyl-functionalized MWCNTs was used to determine chlorpromazine in tablets and human urine, yielding recoveries of 94.80–101.87% [134]. In contrast, a bulk-synthesized MIP for metribuzin was physically combined with MWCNTs and incorporated into a carbon-paste electrode. The resulting composite showed an improved response compared with the unmodified electrode and achieved recoveries of 81.96–91.60%, with an RSD of 2.5% [60]. These examples illustrate that the integration strategy determines how the conductive nanomaterial interacts with the recognition layer and the electrode surface.

The choice between direct surface polymerization and physical mixing depends on the relative importance of interfacial organization, fabrication simplicity, and control over the contact between the MIP and the conductive support. MIP–MWCNT systems have been applied to the determination of pharmaceuticals, pesticides, and biomolecules, predominantly using electrochemical transduction. Their performance depends not only on the intrinsic properties of the nanotubes, but also on MIP loading, nanotube functionalization, and the resulting organization of the recognition layer at the electrode interface [133].

Table 4 also summarizes the analytical performance of MIP–MWCNT-based sensors, including response time, linear range, limit of detection (LOD), and sensitivity. Regarding response time, the insulin sensor was the only device for which this parameter was reported, exhibiting a value of 15 s. The linear ranges varied considerably among the devices, from 0.001 µM for metribuzin to 380 µM for gallic acid. Concerning the limits of detection, the insulin sensor showed the lowest value (1.86 × 10^−5^ µM), followed by the chlorpromazine (0.000296 µM) and metribuzin (0.0005 µM) sensors.

#### 4.1.4. Graphene Oxide

Graphene oxide (GO) has been widely incorporated into MIP-based sensing systems because of its high specific surface area, abundant oxygen-containing functional groups, and good dispersibility in aqueous media. These surface functionalities facilitate interactions with polymer matrices and provide sites for further chemical modification, making GO versatile support for the formation and organization of imprinted layers [138,139].

The role of GO in MIP-based sensors depends on how it is integrated into the sensing interface. GO can provide high-surface-area support for the recognition layer and contribute to interfacial organization through its surface functional groups. In electrochemical systems, partial reduction in GO or its combination with other conductive materials can additionally improve charge transport. In contrast, pristine GO has limited electrical conductivity because oxidation disrupts the conjugated structure of graphene [140]. Therefore, the chemical state of the graphene-based material is an important factor when GO is incorporated into electrochemical sensing interfaces.

Different strategies have been investigated for integrating GO with MIPs, including incorporation of GO into the polymerization mixture, direct formation of MIP layers on GO-modified electrodes, and physical combination of pre-synthesized MIPs with graphene-based materials [141,142]. These approaches can result in different degrees of contact between the MIP, GO, and transducer, affecting recognition-site accessibility and charge transport pathways. The type and extent of GO functionalization, together with the amount and organization of the MIP layer, are therefore relevant parameters for controlling the response of the resulting sensor.

Representative studies illustrate the effect of the integration strategy on analytical performance. Incorporation of GO during MIP synthesis was used to prepare sulfanilamide-selective MIP-GO composites, with the resulting sensor showing a detection limit of 10 ng mL^−1^ and recoveries of 94.8–103.4% in milk samples [140]. In another configuration, GO was first deposited on a glassy carbon electrode, followed by deposition of a pre-synthesized MIP, forming a layered MIP/GO/GCE interface. This architecture provided a detection limit of 1 nmol L^−1^ for 2,4-dichlorophenol and recoveries of approximately 98% in lake water [141]. In contrast, physical mixing of a bulk-synthesized MIP with reduced graphene oxide produced a simpler hybrid film, but with a detection limit of 0.05 µmol L^−1^ for trichloroacetic acid [142]. These examples show that the arrangement of the MIP and graphene-based material relative to the transducer can affect the analytical response. However, direct comparison of detection limits obtained for different analytes and sample matrices should be avoided.

The performance of MIP-GO systems depends on the oxidation state, functionalization, and loading of the graphene-based material, as well as its spatial organization relative to the imprinted layer and transducer. GO may primarily act as structural support, whereas partially reduced or more conductive graphene-based materials can additionally contribute to charge transport. The integration strategy therefore determines the role of the graphene-based component in the sensing interface [143].

**Figure 5:**
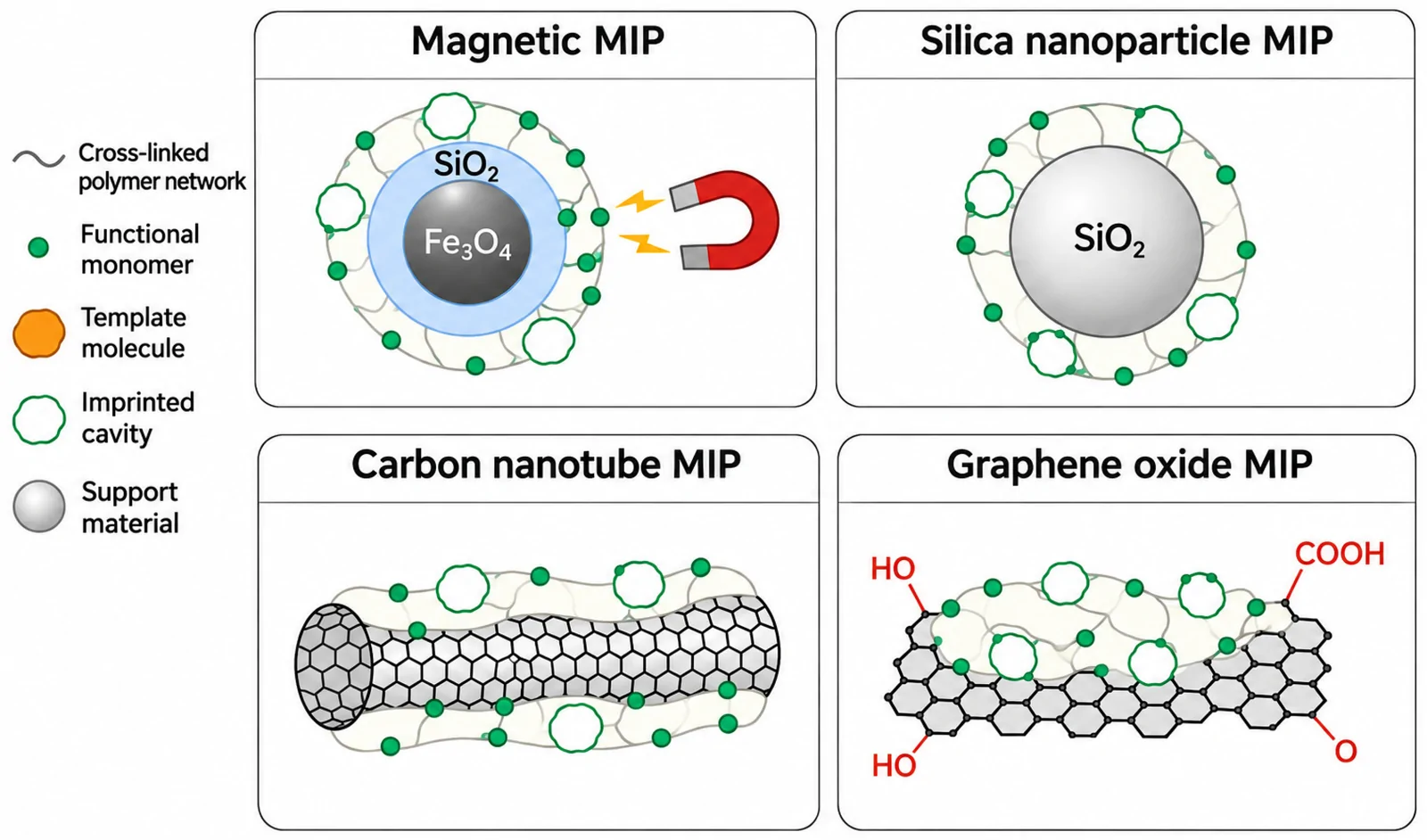
Schematic representation of established support-based MIP architectures, including magnetic, silica nanoparticle-, carbon nanotube-, and graphene oxide-based systems.

Representative examples of support-based MIP architectures, including magnetic, silica nanoparticle-, carbon nanotube-, and graphene oxide-based systems, are schematically illustrated in Figure 5.

### 4.2. Emerging MIP Architectures

**Figure 6:**
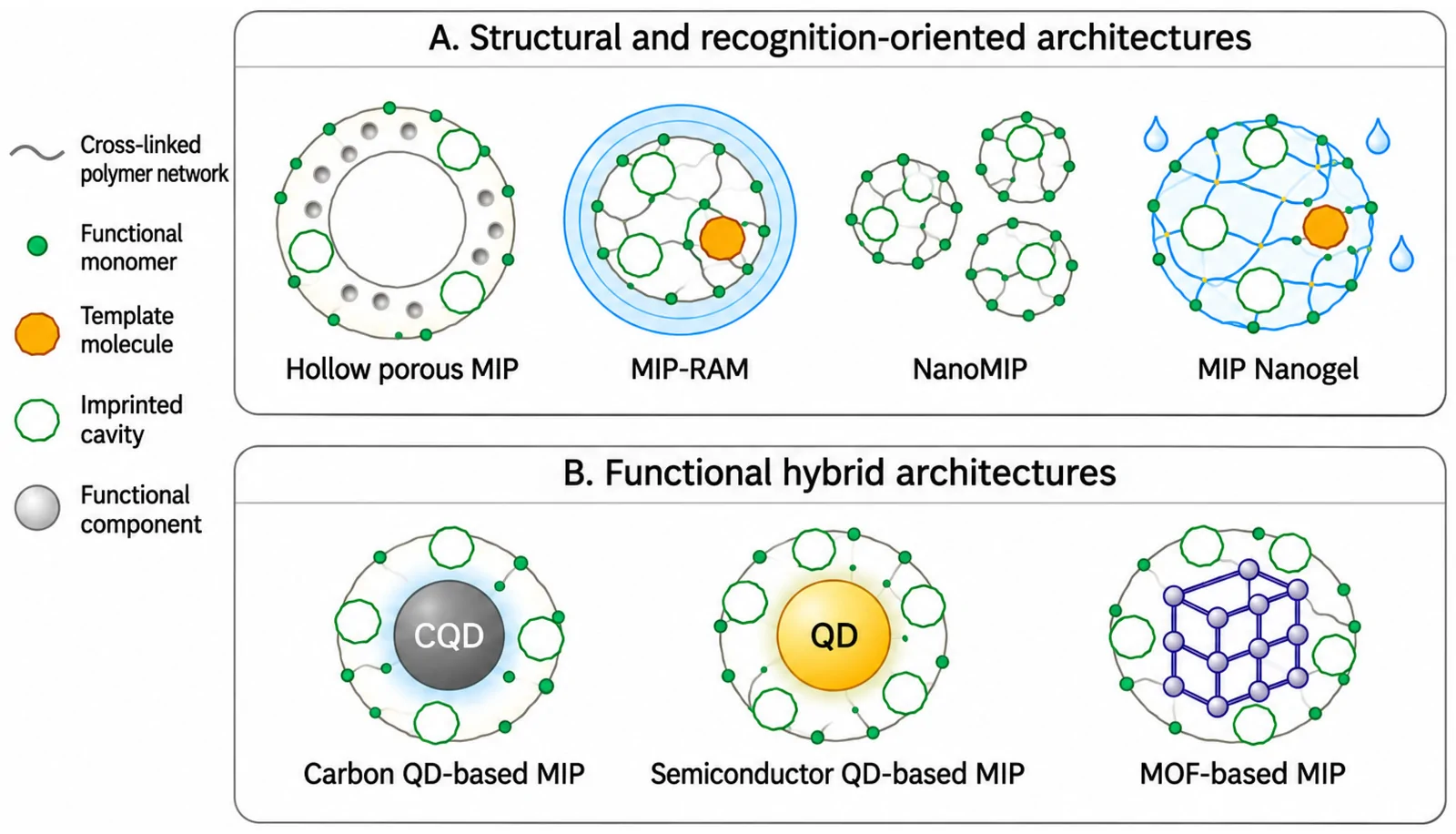
A schematic representation of emerging MIP architectures, including hollow porous MIPs, restricted-access MIPs (MIP-RAM), nanoMIPs, molecularly imprinted nanogels, and hybrid systems incorporating carbon quantum dots, semiconductor quantum dots, and metal–organic frameworks.

Emerging MIP architectures and hybrid systems have been developed to overcome limitations associated with conventional bulk and support-based materials and to introduce additional functionalities into molecular recognition systems. These approaches include modifications to the internal or surface structure of the imprinted polymer to improve analyte transport, recognition-site accessibility, and resistance to matrix interference, as well as the incorporation of functional materials that provide complementary structural, optical, electronic, or physicochemical properties. The following sections discuss representative examples, including hollow porous MIPs, restricted-access MIPs, nanoMIPs, molecularly imprinted nanogels, and hybrid systems incorporating quantum dots and metal–organic frameworks. The main structural and functional hybrid architectures discussed in this section are schematically summarized in Figure 6.

#### 4.2.1. Hollow Porous MIPs

Hollow porous molecularly imprinted polymers (H-MIPs) have been developed to overcome diffusion and accessibility limitations associated with conventional bulk MIPs. These materials typically consist of a hollow interior surrounded by a porous polymer shell, providing accessible inner and outer surfaces for interaction between the analyte and the recognition sites. Hollow architectures are commonly prepared using sacrificial cores, particularly mesoporous silica nanoparticles, whose high surface area, tunable pore structure, and well-defined morphology facilitate the formation of porous imprinted shells [144].

The combination of hollow cavities, interconnected porosity, and accessible internal and external surfaces can facilitate analyte diffusion and increase the fraction of recognition sites available for binding. These characteristics can improve binding kinetics and accessible binding capacity compared with conventional bulk MIPs or solid core–shell MIPs [145,146].

The hollow architecture has also been incorporated into different sensing configurations. Hollow MIPs prepared using a sacrificial SiO_2_ core have been applied to fluorescence sensing of 2,4,6-trichlorophenol, with recognition sites distributed on both the inner and outer surfaces of the imprinted material [146]. More recently, hollow architectures have been combined with functional inorganic and carbon-based components, extending their application beyond the control of analyte transport [147]. Examples include hollow structures integrated with quantum dots and MOFs for ratiometric fluorescence sensing, and hollow mesoporous carbon architectures combined with conductive polymers for electrochemical sensing [148].

In these hybrid systems, the hollow component can contribute directly to signal generation or charge transport, while the MIP provides the molecular recognition sites. A representative example is a hollow ZnS-CdS nanocage combined with an electropolymerized *o*-phenylenediamine MIP film for the photoelectrochemical detection of oxytetracycline. In this architecture, the hollow ZnS–CdS nanocage provided photoactive and charge transfer properties, whereas the MIP supplied the selective recognition sites. The resulting sensor showed a clear difference between the MIP and NIP responses and selectivity toward structurally related interferents. It also showed an inter-electrode RSD of approximately 1.43%, retained 91.5% of its initial response after 30 days at 4 °C, and provided recoveries of 96.6–108.5% in tap and river water [149].

Hollow MIPs have also been investigated as selective sorbents for sample preparation. A hollow MIP for tetracycline showed greater surface area, pore volume, and extraction efficiency than conventional and core–shell MIPs and achieved recoveries of 74–96% in water, synthetic urine, and milk samples prior to HPLC-UV analysis [150]. More recently, a xylose-selective hollow porous MIP further illustrated the potential of this architecture for selective extraction from complex matrices. The material showed an adsorption capacity of 22.99 mg g^−1^, more than twice that of a conventional MIP, together with an adsorption efficiency of 83.80% in sugarcane bagasse hydrolysate and an analyte recovery above 93% after three reuse cycles [151].

Despite these advantages, hollow MIPs generally require more elaborate fabrication procedures than conventional bulk materials, particularly when sacrificial cores must be prepared, coated, and subsequently removed. Control over shell thickness, pore structure, and core removal is important because these parameters affect recognition-site accessibility and the mechanical stability of the final material. In hybrid systems, the incorporation of conductive, optical, or porous components introduces additional interfaces among the hollow scaffold, the imprinted layer, and the transducer, increasing the number of variables that must be controlled to obtain reproducible performance [152].

#### 4.2.2. MIP-RAM

Restricted-access molecularly imprinted polymers (MIP-RAMs) combine molecular recognition with restricted access to the imprinted sites, addressing interference caused by macromolecules in complex samples. These materials typically incorporate a hydrophilic or otherwise size-selective outer layer that limits the adsorption and penetration of macromolecules, such as proteins, while allowing smaller target analytes to reach the imprinted binding sites. This architecture is particularly relevant when the recognition material is directly exposed to biological or other complex matrices [153].

The restricted-access layer can reduce non-specific adsorption of matrix components and limit surface contamination and biofouling, while the underlying MIP provides selective interactions with the target analyte. However, the barrier must be sufficiently permeable to allow the target molecule to reach the recognition sites. Excessive restriction can hinder analyte transport and increase the time required for binding. Therefore, the permeability and physicochemical properties of the outer layer must be controlled to provide effective exclusion of macromolecular interferents without compromising analyte diffusion and binding kinetics [154].

MIP-RAM architectures are particularly relevant to biological and environmental samples, in which matrix components can interfere with molecular recognition and signal generation. By combining matrix exclusion and molecular recognition within the same material, these systems can reduce the need for extensive sample pretreatment and facilitate the use of MIPs in complex samples [153].

A different strategy for reducing matrix effects has recently been reported by Karrat et al. Rather than restricting access to the recognition sites through an external barrier, the study focused on reducing non-specific interactions at the exposed MIP surface. Density functional theory (DFT)-guided monomer selection was combined with covalent hydrophobic surface functionalization to develop an MIP-based colorimetric sensor for sulfamethoxazole [155]. Oleic-acid functionalization resulted in an MIP with higher selectivity and adsorption capacity toward sulfamethoxazole, whereas the corresponding NIP and structurally related analogs showed minimal binding. This approach illustrates an alternative strategy for controlling matrix effects by modifying the surface chemistry of the MIP rather than introducing a restricted-access layer.

These findings show that control of non-specific interactions depends not only on the molecular imprinting process but also on the physicochemical properties of the MIP and its interaction with the sample matrix. In MIP-RAM systems, the main challenge is to obtain sufficient exclusion of macromolecular interferents while maintaining target transport to the recognition sites. Surface functionalization strategies provide an alternative when non-specific adsorption at the exposed polymer surface is the dominant limitation.

#### 4.2.3. NanoMIPs and Solid-Phase Imprinting

Molecularly imprinted nanoparticles (nanoMIPs) have been developed to increase the accessibility of recognition sites and reduce the diffusion limitations associated with highly crosslinked bulk polymers. Because of their small dimensions, a larger fraction of the recognition sites can be located close to the particle surface, facilitating analyte transport and interaction with the imprinted sites [156]. NanoMIPs can be prepared using different polymerization strategies, including precipitation, emulsion, suspension, and other approaches for controlling particle formation. However, reducing the particle dimensions does not necessarily result in a homogeneous population of recognition sites. Particle size distribution, surface chemistry, aggregation, and differences in site accessibility can still contribute to heterogeneous binding behavior.

Solid-phase imprinting has been developed as an alternative approach for improving control over nanoMIP preparation. In this strategy, the template is first immobilized on a solid support, and polymerization is subsequently performed around the surface-bound template. After polymer formation, the nanoparticles are removed from the support and the template is removed from the polymer, generating nanoparticles with recognition sites complementary to the immobilized template [94]. Immobilization changes the way in which the template is presented during polymerization and can be particularly useful for large or structurally complex molecules, for which conventional imprinting in homogeneous solution can be less controlled.

An additional advantage of solid-phase imprinting is the possibility of fractionating the resulting nanoparticles according to their interaction with the immobilized template. Rather than treating all particles obtained from the synthesis as a uniform receptor population, fractionation can be used to obtain populations with different binding characteristics [157]. The immobilized template can also be recovered and reused, which may be relevant when expensive biological templates are employed. Thus, solid-phase imprinting introduces a degree of control over the preparation and selection of nanoMIPs that is not necessarily achieved by conventional nanoparticle synthesis.

However, solid support is not an inert component of this process. Its surface chemistry, morphology, and interaction with the template can affect template density and orientation and, consequently, polymer growth and formation of the recognition sites. This effect was demonstrated in recent experiments comparing glass beads and magnetic nanoparticles as solid supports. Although the same initial monomer formulation was used, the choice of support affected particle size, yield, polymer composition, and binding affinity [156,158]. These results indicate that the solid phase itself is an important parameter in nanoMIP synthesis and that changing the support can alter the resulting recognition material even when the nominal polymerization formulation remains unchanged.

For sensing applications, nanoMIPs can be immobilized on electrodes, optical substrates, or other transducers, allowing for the formation of thin recognition layers with a relatively high density of accessible binding sites [156]. However, the advantages associated with nanoscale dimensions are accompanied by additional variables related to particle size distribution, surface chemistry, aggregation, template density, and immobilization at the transducer. Therefore, nanoMIPs should not be considered simply as smaller versions of conventional MIPs. Their preparation introduces additional parameters that can determine the number, accessibility, and binding affinity of the recognition sites and consequently affect the analytical response.

#### 4.2.4. MIP Nanogels

Molecularly imprinted nanogels are nanoscale polymer networks with a high degree of hydration and relatively low crosslinking density. Their structure differs from the more rigid networks typically obtained in conventional MIPs, allowing for changes in particle volume and polymer conformation in response to the surrounding medium. These changes depend on parameters such as monomer composition, crosslinking density, and the presence of responsive groups. The hydrated nature of nanogels can be particularly relevant for molecular recognition in aqueous and biological media, especially when the target molecules are large or structurally flexible [159].

The small dimensions of nanogels reduce the diffusion distance between the external medium and the recognition sites, while the hydrated polymer network facilitates analyte transport through the material. These characteristics are particularly relevant for the recognition of peptides and proteins, for which diffusion through conventional highly crosslinked MIPs and subsequent template removal can be difficult [160]. However, the same flexibility that facilitates swelling can also modify the environment of the recognition sites and, consequently, the interaction with the target molecule. The recognition properties of nanogels are therefore dependent not only on the chemical interactions introduced during imprinting, but also on the physical state of the polymer network.

Thermoresponsive nanogels provide an example of how these structural changes can be used in sensing systems. Monomers such as N-isopropylacrylamide (NIPAm) can induce temperature-dependent changes in polymer hydration and conformation, which can modify the accessibility of the recognition sites and the interaction with the target molecule [159]. These changes can also be used to facilitate target release or modify the interaction between the recognition material and the transducer. In this case, the nanogel does not function only as a recognition material, but also as a polymer network whose physical state can be controlled during the analytical process.

Recent studies have applied MIP nanogels to biomolecular recognition. Epitope-imprinted nanogels prepared using peptides derived from periodontal-disease biomarkers showed nanomolar affinity and selective recognition in complex bacterial media, demonstrating the applicability of hydrated nanoscale networks to protein-related targets [161]. More recently, epitope-imprinted nanogels prepared by solid-phase synthesis in aqueous media were integrated with screen-printed electrodes for the detection of cardiac troponin T, combining epitope imprinting with electrochemical transduction [162].

The use of nanogels also introduces additional variables that must be controlled during synthesis and sensing. Crosslinking density, particle size, swelling state, and the composition of the surrounding medium can modify the accessibility and affinity of the recognition sites. Changes in temperature, ionic strength, pH, and solvent composition can therefore affect the analytical response. In addition, control of particle size distribution, surface functionality, crosslinking, and batch-to-batch composition is required to obtain reproducible recognition properties [159].

The reproducible application of MIP nanogels in sensing therefore requires control over network composition, swelling behavior, recognition-site heterogeneity, and stability [159].

#### 4.2.5. Carbon Quantum Dots

Carbon quantum dots (CQDs), including graphene quantum dots (GQDs) and heteroatom-doped carbon dots, have been incorporated into MIP-based sensing systems because of their photoluminescence, small size, aqueous dispersibility, and surface chemistry [163,164,165]. Their surfaces contain oxygen-containing groups and, depending on the synthesis and doping procedure, nitrogen, sulfur, or other heteroatoms. These groups can interact with the polymer matrix and provide sites for further surface functionalization.

The optical properties of CQDs can be modified by heteroatom doping and by their incorporation into hybrid structures containing metal oxides, magnetic materials, or other functional components [166]. Heteroatom doping can modify the electronic structure and surface states of carbon dots, thereby affecting their photoluminescence. The incorporation of magnetic or inorganic components can additionally introduce properties such as magnetic manipulation or charge transfer pathways. These modifications can enhance the functionality of the signal-generating component without replacing the molecular recognition provided by the MIP [167,168].

The distinction among pristine CQDs, heteroatom-doped CQDs, and GQDs is relevant because their electronic structures, surface chemistry, and photoluminescence properties are not identical. These differences should be considered when selecting and incorporating carbon-based quantum dots into MIP architectures, as they influence the properties and functionality of the resulting hybrid materials [163].

#### 4.2.6. Semiconductor Quantum Dots (QDs)

Semiconductor quantum dots (QDs) are nanoscale semiconductor crystals whose optical and electronic properties depend on particle size because of quantum confinement. Their tunable absorption and emission, narrow emission bands, broad excitation ranges, and high photostability make them attractive functional components for incorporation into MIP-based sensing architectures [169,170].

Different architectures have been investigated for combining QDs with MIPs. These include direct formation of the imprinted layer on functionalized QDs, incorporation of QDs into silica or other porous supports before MIP formation, and combination of QDs with magnetic or carbon-based materials [94,158]. These configurations determine the spatial relationship between the recognition sites and the QDs and can affect analyte accessibility and the overall functionality of the resulting hybrid material. Core–shell architectures can be particularly useful when the QD requires protection from the surrounding medium while maintaining sufficient interaction with the imprinted layer.

The composition of the quantum dot is an important factor in these systems. Cadmium- and lead-containing QDs can provide suitable optical properties but raise concerns regarding the use of potentially toxic elements. This has motivated the investigation of alternative compositions with improved safety profiles [171]. Changing the QD composition, however, can also modify its emission properties, surface chemistry, stability, and interaction with the MIP. Therefore, the choice of QD must consider not only its intrinsic properties but also its chemical stability, surface functionalization, compatibility with the imprinted layer, and suitability for the intended application.

#### 4.2.7. Metal–Organic Frameworks

Metal–organic frameworks (MOFs) are coordination networks assembled from metal centers and multidentate organic linkers, often featuring well-defined porous structures [172]. Their framework composition, surface chemistry, and accessible porosity can be exploited to integrate MIPs into sensing architectures [173,174,175,176,177]. Depending on the system, the MOF can support the MIP layer, contribute to analyte preconcentration, or participate directly in signal generation. The resulting hybrid therefore combines the molecular recognition properties of the MIP with the structural and physicochemical properties of the MOF [178].

In MOF–MIP systems, the distribution of the polymer within the MOF structure is an important factor. Surface imprinting can position recognition sites at accessible regions of the framework, whereas excessive polymer growth may cover the MOF surface or block its pores. In this case, increasing the amount of MIP does not necessarily improve the performance of the hybrid material, since the resulting reduction in accessible porosity can hinder analyte transport [179]. The amount and location of the imprinted polymer must therefore be considered together with the pore structure of the MOF.

MOFs and MIPs can be combined by forming an imprinted coating on MOF surfaces, incorporating MOF particles into an imprinted polymer matrix, or depositing an imprinted film on a MOF-containing electrode [180,181]. These approaches may overlap and produce different interfaces between the two materials. In surface-imprinted systems, the MOF provides porous support for the formation of accessible recognition sites. Dispersed MOF particles form interfaces throughout the polymer matrix and may contribute to analyte uptake or signal transduction, while the polymer provides selective recognition. In electropolymerized systems, the imprinted film is formed directly on the MOF-containing electrode, whose composition can influence analyte accumulation, charge transport and consequently the electrochemical response [182,183].

The function of the MOF therefore depends strongly on its composition, physicochemical properties, and position within the sensing architecture. In some systems, its main contribution is related to porosity and analyte accessibility, whereas in others it also participates in signal generation or charge transport. This distinction is important because the presence of a MOF does not necessarily mean that the hybrid material will provide better analytical performance. The contribution of the MOF should be evaluated in relation to the MIP and, when applicable, to the additional material used for signal transduction [184].

MOF–MIP systems also introduce limitations associated with the stability and reproducibility of the hybrid structure. The stability of the MOF can depend on solvent composition, pH, ionic strength, temperature, and exposure time. Framework integrity should therefore be evaluated under the conditions used during polymerization, template removal, sensing, and regeneration. At the same time, MIP formation can modify the surface of the framework and partially block its pores. Additional variables, including MOF crystal size, surface functionalization, MIP loading, and layer thickness, can affect the final response. Control of these parameters is necessary to distinguish the contribution of the MOF from that of the MIP and to ensure reproducible sensing performance [184]. Representative MOF–MIP-based sensing architectures, their integration strategies, and corresponding transduction modes are summarized in Table 5.

Beyond structural stability, the practical applicability of MOF–MIP materials also depends on their regeneration and reusability. Template removal during MIP preparation, which exposes the recognition sites, should be distinguished from regeneration during analytical operation, which involves removing previously bound analytes to restore the material’s recognition capacity. Regeneration performance should be evaluated by monitoring signal recovery, baseline stability, and response retention over successive sensing cycles. Therefore, successful template removal or evidence of material stability alone cannot establish sensor reusability [196].

Although conventional preparation methods and advanced MIP architectures represent different levels of material design, both influence key properties relevant to sensing performance. Conventional methods primarily determine particle morphology, size distribution, and porosity, whereas advanced architectures introduce additional structural and functional features that can modify recognition-site accessibility, analyte transport, and transducer integration. Consequently, the selection of a suitable MIP system should consider not only the preparation method or architecture individually, but also how these characteristics contribute to the requirements of the intended sensing application.

The conventional preparation methods and advanced architectures discussed in Section 3 and Section 4 differ in their ability to control material morphology, provide accessible recognition sites, facilitate analyte transport, and support integration with sensing platforms. Their main characteristics and implications for sensing applications are summarized in Table 6.

## 5. MIP-Based Sensor Platforms

MIPs can be integrated into different sensor platforms, in which molecular recognition events are converted into measurable analytical signals. Among the most widely investigated approaches, electrochemical and optical transduction offer complementary strategies for MIP-based sensing. Electrochemical platforms convert analyte recognition into changes in current, potential, charge transfer, or interfacial properties, whereas optical platforms rely on variations in optical signals, including fluorescence, light absorption, Raman scattering, and surface plasmon resonance.

Electrochemical transduction has received considerable attention in the development of MIP-based sensors, resulting in a broader range of reported architectures and analytical applications. Nevertheless, optical approaches also offer important opportunities for molecular recognition and chemical sensing, particularly through fluorescence, surface-enhanced Raman scattering (SERS), and surface plasmon resonance (SPR) platforms. Their relative representation in this review reflects the current distribution of the literature rather than a limitation in the potential of optical MIP-based sensors.

**Figure 7:**
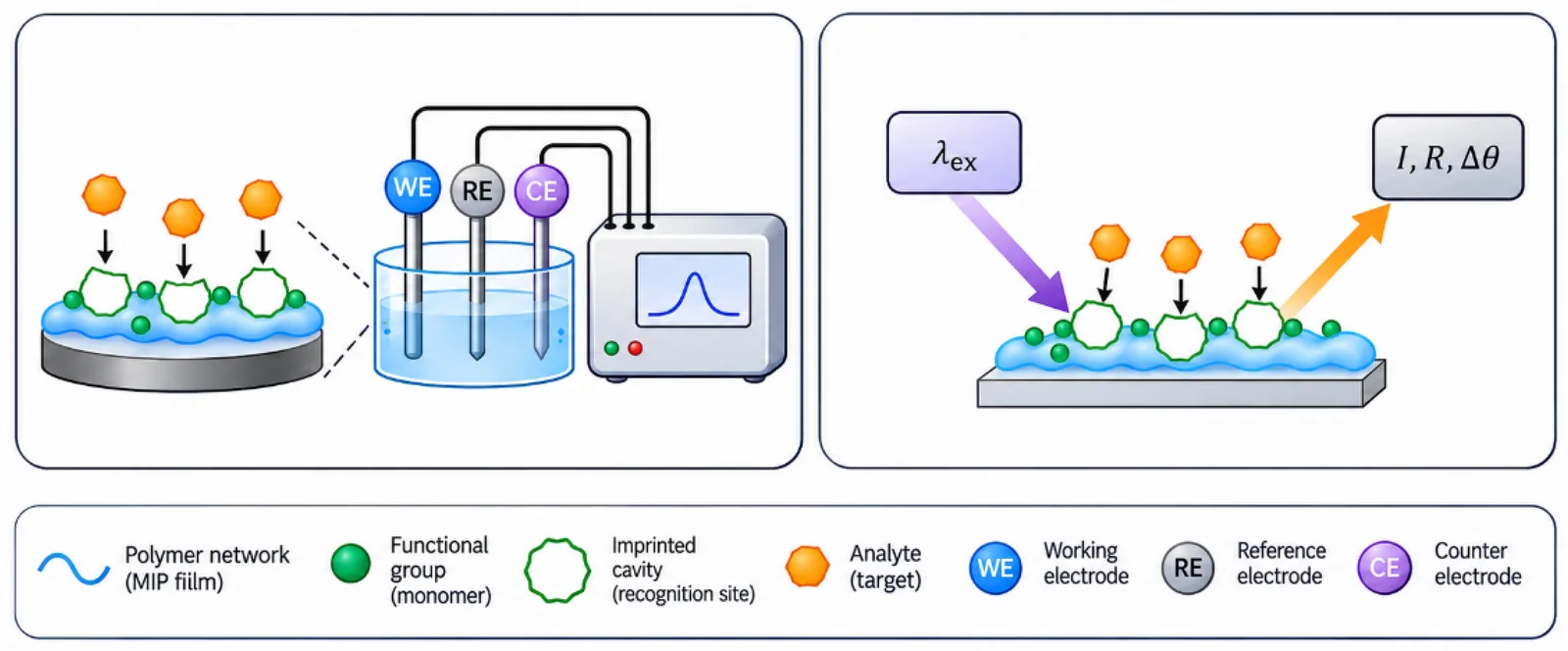
A schematic representation of representative electrochemical and optical MIP-based sensor configurations, illustrating molecular recognition at the imprinted polymer layer and the corresponding signal transduction mechanisms.

Beyond the transduction mechanism, the practical performance of these platforms depends on several factors, including sensitivity, linear or calibration range, response time, selectivity, operational stability, and regeneration efficiency. These parameters are considered throughout the following sections whenever the corresponding information is available in the reported studies. The main characteristics and representative applications of electrochemical and optical MIP-based sensor platforms are discussed in Section 5.1 and Section 5.2, respectively. Representative configurations of electrochemical and optical MIP-based sensors, illustrating the integration of the recognition layer with the respective transduction systems, are schematically presented in Figure 7.

### 5.1. MIP-Based Electrochemical Sensor Platforms

#### 5.1.1. MIP Synthesis by Electropolymerization

Electropolymerization is widely used for MIP preparation because the polymer can be formed directly on the electrode surface, allowing for control over the amount of deposited material and the coverage of the transducer. Compared with polymerization methods in which the MIP is prepared separately, electropolymerization simplifies the fabrication of the recognition layer and is particularly suitable for modified, miniaturized, and disposable electrodes [20]. The direct formation of the polymer at the electrode also allows the thickness and morphology of the recognition layer to be adjusted according to the electrochemical conditions used during polymerization.

The properties of the resulting MIP film depend strongly on the functional monomer and on the electrochemical behavior of the polymer formed. Monomers such as pyrrole (Py), aniline (ANI), thiophene derivatives, and 3,4-ethylenedioxythiophene (EDOT) can produce electroactive, potentially conductive polymer films, whereas monomers such as *o*-phenylenediamine (o-PD), phenol derivatives, and aminophenols primarily form thin, insulating recognition layers [197,198]. The classification of monomers as conductive or non-conductive is related to the electrical conductivity characteristics of the polymer formed after electropolymerization, that is, its ability to produce conductive or insulating polymer films. However, the conductive or non-conductive nature of the resulting MIP can be influenced by the doping level, the potential window applied to the sensor, the pH and electrolyte composition, as well as post-polymerization treatments [197,198]. ANI, for example, forms a polyaniline (PANI) polymer film that exhibits conductive properties in its protonated emeraldine salt form but loses much of its conductivity at pH values close to neutrality [197,199]. On the other hand, the overoxidation of polypyrrole (PPy) or PANI, frequently employed for template removal, introduces carbonyl groups and disrupts the conjugated backbone, thereby modifying the electrical and electrocatalytic properties of the film [197,199]. Therefore, in this review, electropolymerized MIPs are grouped according to the behavior of the polymer under the reported operating conditions and its contribution to signal transduction, rather than being classified exclusively according to the monomer name. In conductive systems, the polymer can directly contribute to charge transport at the electrode interface. In contrast, non-conductive MIPs generally act as selective barriers, in which the interaction with the analyte modifies the accessibility of the electrode surface to either the analyte itself or an external redox probe [198,200].

This distinction also determines how the molecular recognition event is converted into an electrochemical response. In conductive MIPs, the target can generally be monitored directly when the analyte is electroactive, and the polymer architecture allows it to interact with the electrode. For non-conductive films, detection is commonly performed indirectly by monitoring changes in the response of a redox probe, such as the ferri/ferrocyanide pair, since target binding modifies the permeability or interfacial properties of the MIP layer [201]. Therefore, the electrical properties of the polymer are directly related to the mechanism by which recognition is transduced and should be considered during the design of the MIP film.

A third configuration, increasingly explored for portable and wearable devices, consists of electropolymerized MIPs with an embedded redox mediator. A redox-active species, such as Prussian blue nanoparticles or anthraquinone derivatives, is incorporated within or beneath the imprinted layer, so that target binding modulates the mediator signal without adding a probe to the sample [202,203]. A cortisol-imprinted polypyrrole film containing Prussian blue, for example, enabled label-free amperometric detection of cortisol in fingertip sweat in about 3.5 min [202]. Similarly, laser-engraved graphene electrodes carrying Prussian blue nanoreporters beneath pyrrole-based MIPs allowed for the indirect detection of non-electroactive amino acids, vitamins, and metabolites in sweat. These sensors were regenerated in situ by applying a constant potential, maintained stable redox signals over 60 consecutive CV cycles, and showed minimal changes after 42 days of storage [203].

**Figure 8:**
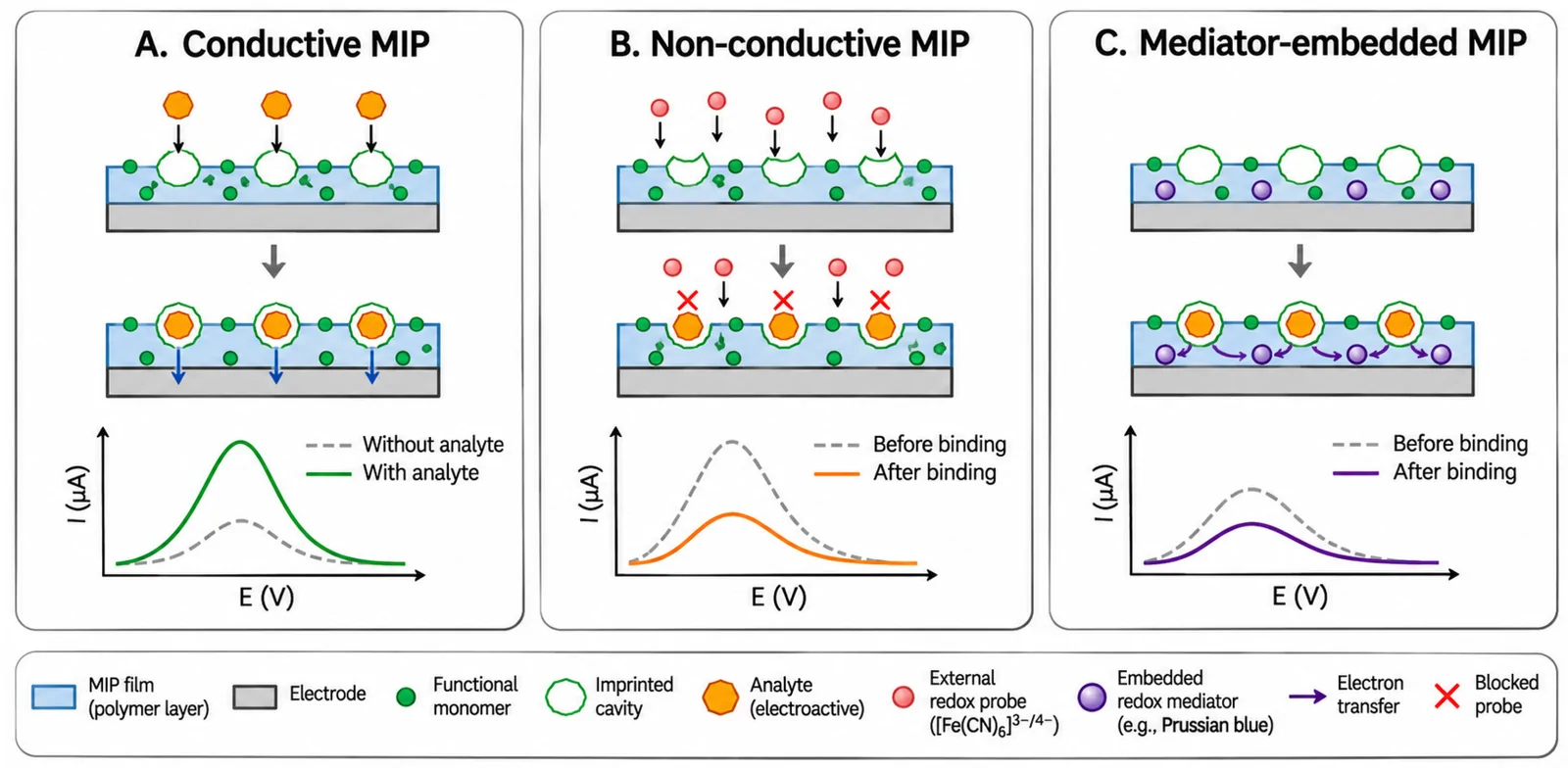
A schematic representation of electrochemical signal transduction mechanisms in electropolymerized MIP films: (**A**) conductive MIPs enabling direct electrochemical detection of electroactive analytes; (**B**) non-conductive MIPs using an external redox probe; and (**C**) mediator-embedded MIPs incorporating redox-active components within the polymer film.

These three configurations involve different compromises. Non-conductive MIPs interrogated with a solution-phase probe such as [Fe(CN)_6_]^3−/4−^ are simple to prepare and often reach low detection limits, because thin, self-limiting films produce large signal changes when binding blocks the electrode surface. However, the probe must be present in every measurement, readings usually follow incubation periods of several minutes, the response is typically inverse and frequently non-linear, and regeneration generally relies on solvent washing. These features favor single-use or laboratory measurements over continuous monitoring. Mediator-embedded MIPs remove the external probe and are compatible with measurements directly in biofluids and with electrochemical regeneration, but their operational and shelf stability depend on the retention and redox stability of the mediator, and their calibration is often log-linear [203]. Intrinsically conductive or electrocatalytically active MIPs allow for direct oxidation of electroactive targets. In a polyaniline-based MIP for phenylalanine in sweat, electrochemical degradation of the polymer during template removal generated electrocatalytic sites, giving a linear range of 10–300 µM, a sensitivity of 1.4 nA µM^−1^, an LOD of 4.7 µM, and low batch-to-batch variation [199]. This direct and positive response is, however, restricted to electroactive analytes and is more sensitive to pH: the sensor was stable only between pH 7.0 and 9.5, which required an in situ buffering layer for measurements in weakly acidic sweat [199]. Regeneration efficiency and operational stability are still rarely reported for conductive and non-conductive electropolymerized MIPs, which hinders a quantitative ranking of these strategies (Table 7 and Table 8). The main electrochemical signal transduction mechanisms associated with these three configurations are schematically illustrated in Figure 8.

Film thickness is an important parameter in both conductive and non-conductive MIPs. Increasing the thickness of the film increases the amount of polymer deposited and may provide a larger number of recognition sites, but it also increases the diffusion path for the analyte and can hinder electron transfer across the interface. Very thin films can reduce these transport limitations but may contain fewer recognition sites. One of the advantages of electropolymerization is the possibility of controlling film growth through parameters such as the applied potential, polymerization time, monomer concentration, and number of electrochemical cycles [204]. These parameters therefore need to be adjusted according to the mechanism used for detection rather than simply maximizing the amount of polymer deposited.

Template removal is another important step in the preparation of electropolymerized MIPs. Depending on the polymer and template, extraction can be performed using appropriate solvents or electrolytes. In conductive polymers such as polypyrrole, overoxidation can also modify the polymer structure and facilitate template release. The removal procedure must provide sufficient extraction of the template without compromising the integrity and adhesion of the film. Incomplete removal can result in template bleeding and residual occupancy of the recognition sites, whereas excessively aggressive treatment can damage the polymer layer and affect reproducibility [205].

Electropolymerized MIPs have also been combined with nanostructured electrode modifiers, including metallic nanoparticles, carbon nanomaterials, graphene derivatives, and MOFs [206,207,208,209,210]. The modifier can increase the electroactive surface area, facilitate charge transfer, and, depending on its composition, provide catalytic or other additional functions. However, the presence of conductive or electroactive material does not necessarily improve the recognition properties of the MIP. Its effect depends on the organization of the MIP layer at the modified electrode and on whether the recognition sites remain accessible after incorporation of the additional material.

Other strategies have been investigated to adapt electropolymerized MIPs to specific analytical requirements, including copolymerization of functional monomers, ratiometric measurements, the use of dummy templates, and covalent attachment of the MIP layer to the electrode surface [20,211,212,213,214,215,216,217]. These strategies become particularly relevant for disposable and portable devices, in which variations in electrode fabrication and polymer deposition can contribute to differences between individual sensors.

Despite the versatility of electropolymerization, several factors can affect the reproducibility of the resulting sensors, including adhesion of the polymer to the electrode, film thickness, template removal, and stability during repeated measurements. These limitations become particularly relevant when moving from laboratory-scale electrodes to disposable or portable devices, where variations in substrate fabrication and polymer deposition can contribute substantially to sensor-to-sensor variability [20].

Table 7 and Table 8 summarize representative electropolymerized MIP sensors based on non-conductive and conductive polymers, respectively, including the analytes, electrode configurations, detection methods, and representative analytical characteristics.

**Table 7:** Representative electropolymerized MIP sensors in which the imprinted film acts predominantly as a non-conductive (insulating or poorly conductive) recognition layer under the reported operating conditions, including analytes, electrode configurations, detection methods, and analytical performance.

Analyte	Samples	Functional Monomer	Electrode	Detection Method	Response Time (min)	Linear Range (µM)	LOD (µM)	Sensitivity	Ref
Hippuric acid	Human urine	o-PD	Modified FTO	DPV	1.3	0.005–0.091; 0.091–0.615	0.0013	189.5 µA µM^−1^	[218]
3-MCPD	Vegetable oil and soy sauce.	ANI ^a^	Au-SPE	DPV	40	0.05–0.5; 0.5–15	0.01	1.42 µA µM^−1^	[206]
VB12	Microcapsules	o-AP	Au-SPE	SWV	15	0.5–10	0.5	-	[219]
Deltamethrin	Tomato, salad, grapefruit, and orange	o-PD	Co_3_O_4_/ITO	CV/EIS	15	0.00282–0.0565; 0.25–3.98	0.00153	13.4 µA nM^−1^	[220]
2,4,6-TCP	Water samples	o-PD ^b^	C-SPE	SWV	NR	0.02–1000	0.0067	0.667 µA µM^−1^	[221]
Chlorpromazine	Biological samples	NA	GCE	SWV	10	0.001–40; 40–900	0.025	0.0332 µM^−1^ (ratio)	[222]
IL-6	Human serum	β-CD/Py ^c^	C-SPE	CV/EIS	30	4.8 × 10^−8^–4.8 × 10^−3^	4.8 × 10^−8^	0.046 dec^−1^ (rel. Rct)	[223]
5-hydroxymethylfurfural	Coffee	PABA	GCE	DPV	45	7.9–55.5	2.95	0.0104 µA µM^−1^	[224]

DPV: differential pulse voltammetry; SWV: square wave voltammetry; EIS: electrochemical impedance spectroscopy; CV: cyclic voltammetry; 3-MCPD: 3-chloropropane-1,2-diol; VB12: vitamin B12; 2,4,6-TCP: 2,4,6-trichlorophenol; IL-6: interleukin-6; FTO: fluorine-doped tin oxide; ITO: indium tin oxide; o-AP: o-aminophenol; PABA: 4-aminobenzoic acid; o-PD: ortho-phenylenediamine; ANI: aniline; Au-SPE: gold screen-printed electrode; C-SPE: carbon screen-printed electrode. β-CD: β-cyclodextrin; Py: pyrrole; NA: nicotinamide; Gr/Au-F: graphene/flower-like gold; Rel. Rct: relative charge transfer resistance; dec: decade of concentration. ^a^ Polyaniline is usually classified as a conducting polymer; in the cited study, the authors attributed the decreasing currents during electropolymerization to the formation of an over-oxidized, poorly conductive polyaniline film. 3-MCPD was quantified from its own oxidation peak. ^b^ The MIP layer was formed by poly(o-PD); the conductive character of this platform arises from the covalently assembled graphene/flower-like gold (Gr/Au-F) layer beneath the MIP. ^c^ Polypyrrole is usually classified as a conducting polymer; in the cited study, the poly(β-CD-Py) film was reported to grow as a passivating, non-conductive layer, and IL-6 was quantified through the [Fe(CN)_6_]^3−/4−^ probe by EIS. Values reported in mass units were converted to µM using the molar masses of IL-6 (21 kDa) and HMF (126.11 g mol^−1^). Response time corresponds to the incubation (rebinding or accumulation) time before measurement.

**Table 8:** Representative electropolymerized MIP sensors based on conductive polymers and nanomaterial-modified electrode interfaces.

Analyte	Functional Monomer	Electrode	Modification	Detection Method	Response Time (min)	Sensitivity	Linear Range (μM)	LOQ (nM)	Ref
Norfloxacin	Py	GCE	black phosphorus nanosheet nanocomposite functionalized with AuNPs	LSV	5	63.97 and 1.122 μA μM^−1^	0.0001–10	0.012	[225]
Catechol	Py	GCE	2D layered titanium carbide (MXene) + amino-functionalized bimetallic organic framework (Fe@Ti-MOF-NH_2_)	SWV	3	3.1586 µA µM^−1^	1.0–4000	540	[226]
Amaranth	Py	GCE	multi-walled carbon nanotubes	LSV	20	14.6674 and 1.9628 µA µM^−1^	0.007–1.00 0.4–17	0.4 0.4	[207]
Tryptophan	Py	GCE	CuCo_2_O_4_ @ biomass-derived carbon	DPV	5	9.6259 and 0.9064 µA µM^−1^	0.01–1 1–40	3	[227]
Nicotine	Py	GCE	Platinum nanoparticles + GO	DPV	30	-	0.01–0.5 0.5–100	15	[228]
Sorafenib	Py	CPE	MOFs (MIL-101(Fe))	ASDPV	10	3.9 µA µM^−1^	4.2 × 10^−5^–7.1 × 10^3^	0.0064	[229]
Histamine	p-ABSA	GCE	L-cysteine + AuNPs	DPV	5	0.120 and 0.053 µA µM^−1^	1.0–40.0 40.0–107.0	600	[230]
Paracetamol	Cz and Py	GCE	3D graphene–carbon nanotubes functionalized with ionic liquid + gold–palladium alloy nanoparticles	DPV	4	2.06 μA/μM·mm^2^	0.10–10	50	[231]
Limonene	Py	GCE	carbon nanotubes	DPV	15	0.92 nA L pmol^−1^	1 × 10^−6^– 1 × 10^−4^	8.7 × 10^−4^	[232]

LSV: linear sweep voltammetry; SWV: square wave voltammetry; DPV: differential pulse voltammetry; ASDPV: anodic stripping differential pulse voltammetry; Py: pyrrole; p-ABSA: p-aminobenzenesulfonic acid; Cz: carbazole.

Electropolymerized MIP-based electrochemical sensors have been investigated for the detection of various classes of substances, including pesticides, food contaminants, environmental pollutants, pharmaceuticals, and biomarkers [206,208,218,219,220,221,222,223,224,225,226,227,228,229,230,231,232]. When comparing sensors based on MIPs prepared by electropolymerization using conductive and non-conductive monomers, similar performance was observed in terms of selectivity, linear range, and limit of detection (LOD). However, sensors based on non-conductive monomers exhibited longer response times, as shown in Table 7 and Table 8, because these configurations generally require longer incubation periods than sensors prepared using conductive monomers. In the study by Aryan et al., an electrochemical sensor based on an electropolymerized MIP was developed using the non-conductive functional monomer o-phenylenediamine (o-PD) for the detection of hippuric acid. The sensor achieved a limit of detection (LOD) of 0.13 pmol L^−1^ using a fluorine-doped tin oxide (FTO) electrode modified with ultrafine gold nanoparticles (AuNPs) and graphitic carbon nitride (g-C_3_N_4_) [218]. The linear ranges reported for this type of sensor can vary considerably, extending from concentrations in the mmol L^−1^ range down to the pmol L^−1^ range. For example, Lu et al. reported a linear range from 1 µmol L^−1^ to 4 mmol L^−1^ for catechol detection [226]. In another study, Zancan et al. obtained a calibration curve over the range of 1–100 pmol L^−1^ for limonene determination [232]. Ferreira et al. developed an electrochemical biosensor based on an electropolymerized molecularly imprinted polymer (MIP) for the quantification of interleukin-6 (IL-6) in human serum samples. The sensor exhibited excellent performance in recovery assays using spiked samples and in comparisons with the non-imprinted polymer (NIP), demonstrating the high selectivity provided by MIPs in complex biological matrices [223].

#### 5.1.2. Electrode Substrates and Formats in MIP-Based Sensors

The electrode substrate provides an interface at which the interaction between the analyte and the MIP is converted into an electrochemical response. Its surface chemistry, conductivity, electroactive area, and compatibility with surface modification can affect charge transfer, film adhesion, background currents, and the accessibility of the recognition layer [25]. Therefore, the electrode cannot be considered independently of the MIP architecture and detection mechanism, since the same recognition layer can produce different responses depending on the characteristics of the electrode surface.

Carbon-based electrodes, particularly glassy carbon electrodes (GCEs) and carbon paste electrodes (CPEs), are widely used in MIP-based electrochemical sensors because of their chemical stability, broad potential window, relatively low cost, and compatibility with surface modification [233,234]. GCEs provide a well-defined and mechanically stable surface for deposition of MIP films and nanostructured modifiers [233]. CPEs, in contrast, allow MIPs, nanoparticles, and other components to be incorporated directly into the electrode material, providing greater flexibility in electrode composition. However, variations in paste composition and surface renewal can affect the reproducibility of CPE-based sensors [234].

Screen-printed electrodes (SPEs) differ from GCEs and CPEs not only in composition but also in format. Their planar configuration, low sample consumption, and suitability for mass production facilitate the development of portable and disposable MIP-based sensors. SPEs can also be modified by electropolymerization or by deposition of nanostructured materials directly on the printed surface. However, differences in electrode morphology and manufacturing between batches can contribute to variations in the electrochemical response [235].

Gold electrodes provide readily functionalizable surfaces that can be combined with nanostructured materials. Gold nanoparticles can also be incorporated into hybrid interfaces to increase the electroactive area and modify charge transfer pathways [236]. In MIP-based sensors, however, an increase in electroactive area should not be interpreted directly as an improvement in molecular recognition. The response of the modified electrode can result from changes in charge transfer or surface area, independently of the affinity and selectivity of the MIP.

Other electrode materials have been investigated for specific analytical applications. Bismuth-based electrodes, for example, have been explored as alternatives to mercury-based electrodes, particularly for the electrochemical determination of metal ions [237,238]. In these cases, the choice of substrate is primarily related to the electrochemical behavior of the target and the requirements of the measurement, while the MIP provides the molecular or ionic recognition element.

Electrode modification can further influence the properties of the interface at which the MIP operates. Different nanostructured materials, including carbon nanotubes, graphene derivatives, metallic nanoparticles, and MOFs, have been incorporated into or deposited on electrodes before MIP formation, allowing for the development of hybrid interfaces with tailored electrochemical properties [26]. Depending on their composition and organization, these modifiers can affect the electroactive surface area, charge transfer processes, and catalytic activity. However, their contribution should be evaluated using appropriate controls, since improvements in the electrochemical response do not necessarily indicate enhanced molecular recognition.

Within these hybrid interfaces, the electrode modifier and the MIP perform complementary functions: the former contributes to the electrochemical properties of the interface, whereas the latter provides selective molecular recognition. Representative examples include MOF–MIP systems employing electrochemical transduction (Table 5, Section 4.2.7) and nanomaterial-modified electrodes combined with electropolymerized MIPs (Table 7 and Table 8, Section 5.1.1). Evaluating these contributions separately is essential to determine whether improvements in sensor performance arise from changes in the electrochemical interface, molecular recognition, or both.

The choice of electrode format therefore depends on the relationship between substrate properties, MIP immobilization, and the detection mechanism. GCEs are particularly useful for developing and characterizing MIP-based interfaces under controlled laboratory conditions, whereas CPEs provide greater flexibility in electrode composition and SPEs facilitate the fabrication of miniaturized and disposable devices. The relevant consideration is not only the electrochemical response of the substrate but also whether the resulting interface provides stable and accessible recognition sites and reproducible coupling between molecular recognition and the electrochemical signal.

### 5.2. MIP-Based Optical Sensor Platforms

**Figure 9:**
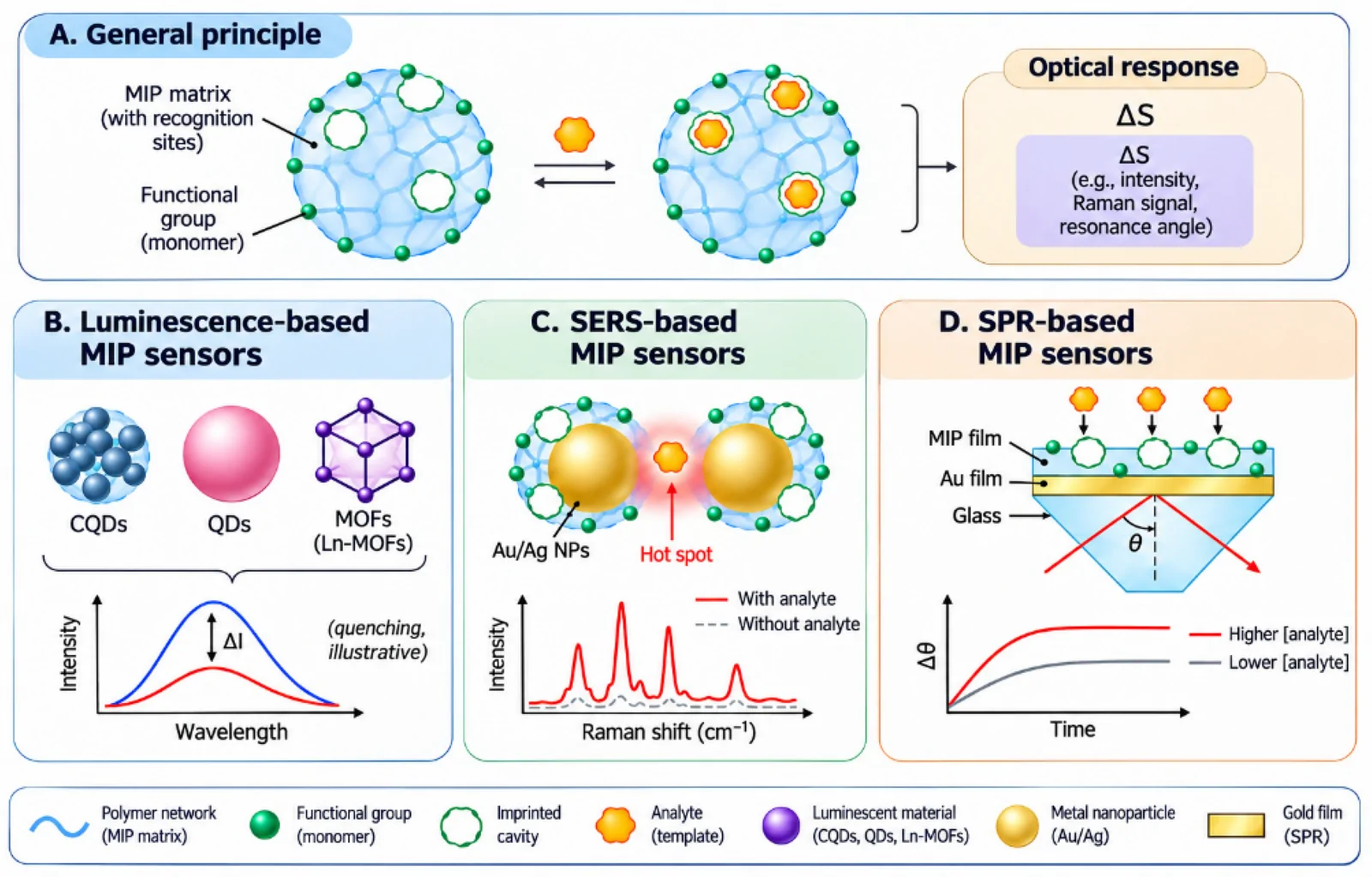
A schematic overview of MIP-based optical sensor platforms: (**A**) general principle of optical detection through analyte recognition; (**B**) luminescence-based sensors using CQDs, QDs, and MOFs; (**C**) SERS-based sensors employing metallic nanoparticles; and (**D**) SPR-based sensors based on changes in the resonance angle upon analyte binding.

MIP-based optical sensors encompass different transduction strategies that convert analyte recognition into measurable optical responses. These include luminescence-based approaches, surface-enhanced Raman scattering (SERS), and surface plasmon resonance (SPR), each relying on distinct optical phenomena and sensing configurations. The general principles and representative architectures of these platforms are schematically illustrated in Figure 9.

#### 5.2.1. Luminescence-Based MIP Sensors

Luminescence-based MIP sensors combine the molecular recognition properties of imprinted polymers with the optical properties of emissive materials, allowing analyte binding to be converted into measurable light signals. Depending on the emissive material and the photophysical processes involved, the optical response may arise from fluorescence or other luminescence mechanisms. Changes in emission intensity, including quenching or enhancement, can be associated with interactions between the target analyte, the imprinted recognition sites, and the luminescent component [239,240]. The efficiency of signal generation therefore depends on the photophysical properties of the emissive material, its spatial organization within the MIP architecture, and the accessibility of the recognition sites [241].

Carbon quantum dots (CQDs), including graphene quantum dots (GQDs), have been incorporated into several MIP-based luminescent sensing systems. CQDs do not provide molecular selectivity by themselves; their incorporation into MIPs therefore combines the photophysical properties of the quantum dots with the molecular recognition provided by the imprinted sites [163]. In these systems, CQDs act as signal-generating components, whereas the MIP selectively interacts with the target analyte. The interaction between these components depends on their surface chemistry and spatial organization, which can affect the accessibility of the recognition sites and the optical response of the composite [242].

The systems summarized in Table 9 illustrate the variety of CQD-based MIP architectures reported for luminescent sensing. Examples include MIPs incorporating N-GQDs for terbuthylazine, ZnO/N-GQD systems for diflunisal, N,S-CQDs for chloramphenicol, GQDs for biotin, and melanin@N-GQDs for prometryn. Other systems combine carbon dots with magnetic or inorganic components, such as NCQDs/SiO_2_, OH-HNTs@N-GQDs@Fe_3_O_4_, and N-GQDs-ZnO/CF-Fe_3_O_4_, introducing additional functionalities into the sensing architecture. These examples demonstrate the versatility of carbon-derived quantum dots as emissive components in MIP-based sensing systems.

In addition to carbon-derived quantum dots, semiconductor quantum dots (QDs) have also been integrated into MIP-based luminescent sensors. Their incorporation allows molecular recognition events to be associated with measurable changes in the emission properties of the quantum dots. Depending on the composition and architecture of the system, target binding can affect the local environment of the QDs, resulting in fluorescence enhancement or quenching [252,253]. The surface of the QD is therefore an important part of the design. Functionalization or encapsulation can improve colloidal stability, provide reactive groups for MIP formation, and protect the emissive material from degradation [254,255].

The examples summarized in Table 10 illustrate the variety of QD-based MIP systems reported for sensing. CdTe-based systems have been used for the detection of malachite green and histamine, whereas CsPbBr_3_ quantum dots have been incorporated into hybrid structures for the determination of rhein and patulin. These examples include combinations of QDs with magnetic or inorganic components, such as nano-Fe_3_O_4_ and SiO_2_ and illustrate the use of different QD compositions and architectures for luminescence-based sensing.

Beyond quantum-dot-based systems, metal–organic frameworks (MOFs) with luminescent properties have also been combined with MIPs to develop optical sensing platforms. In particular, lanthanide-containing MOFs (Ln-MOFs) offer characteristic emission properties that can be exploited in luminescence-based detection. Their integration with MIPs combines the molecular recognition provided by the imprinted sites with the optical properties of the framework. An example is an Eu/Tb-MOF@MIP system developed for smartphone-assisted ratiometric fluorescence detection of perfluorooctanoic acid, in which surface imprinting was combined with the luminescent properties of the Ln-MOF [227]. Other examples include a Zr-LMOF-based fluorescence sensor for chloramphenicol and a Cr-MOF@MIP system for tetracycline detection [190,192]. In a different configuration, a sensor for pyrraline incorporated MOFs and CdSe/ZnS quantum dots. In this system, the QDs provide the fluorescence signal, while the MOF serves as the imprinting matrix [259]. A paper-based LMOF/MIP platform has also been reported for tetracycline detection [193]. Together, these examples illustrate the different roles that MOFs can play in luminescent MIP architectures, acting either as emissive frameworks or as structural components supporting the recognition and signal-generating elements.

The analytical performance of luminescent MOF–MIP systems vary depending on the hybrid architecture, target analyte, and sensing mechanism. For example, the Zr-LMOF-based fluorescence sensor for chloramphenicol exhibited a linear calibration range of 0.0005–0.500 μM and a limit of detection (LOD) of 4.02 × 10^−5^ μM [190], whereas the MOF/CdSe–ZnS QD@MIP system for pyrraline covered a wider concentration range of 5–1000 μM, with an LOD of 3.9 μM [191]. Differences have also been reported among sensors targeting the same analyte. For tetracycline detection, the Cr-MOF@MIP fluorescence sensor exhibited a reported response time of approximately 10 s [192], whereas a paper-based LMOF/MIP platform reported a response time of 30 min [193]. These values should not, however, be interpreted as a direct ranking of sensor performance, since experimental conditions, calibration models, and definitions of response time may differ among studies. Similarly, sensitivity values depend on the analytical signal, the transduction mechanism, and the way sensitivity is defined and calculated. Therefore, calibration range, LOD, response time, and sensitivity should be considered collectively and interpreted in the context of the experimental conditions and characteristics of each sensing system.

#### 5.2.2. Surface-Enhanced Raman Scattering (SERS)-Based MIP Sensors

Surface-enhanced Raman scattering (SERS) amplifies Raman signals near nanostructured metallic surfaces, typically based on gold or silver nanoparticles. This enhancement is mainly attributed to electromagnetic effects associated with localized surface plasmon resonance (LSPR), although chemical enhancement arising from electronic interactions between the analyte and the metallic surface can also contribute [260]. SERS provides highly sensitive molecular fingerprint information, but its selectivity can be limited in complex samples [163,260]. When coupled with MIPs, the recognition layer selectively binds the target analyte and brings it into the vicinity of the SERS-active surface, combining molecular recognition with the chemical information provided by Raman spectroscopy [13,163].

The effectiveness of this combination depends strongly on the spatial relationship between the imprinted layer and the plasmonic surface. Selective binding by the MIP can increase the local concentration of the target analyte near the metallic nanostructures [261,262]. However, if the imprinted layer is too thick, the analyte may remain too far from the electromagnetic hot spots, reducing the contribution of SERS enhancement [262,263]. Conversely, insufficient MIP coverage may result in a lower density of recognition sites and less effective suppression of non-specific adsorption. Therefore, MIP thickness, porosity, and recognition-site distribution must be considered together with the size, geometry, and spatial distribution of the metallic nanostructures [262,263].

Different architectures have been investigated to combine MIPs with SERS-active substrates. These include the incorporation of metallic nanoparticles into the polymer matrix, the deposition of imprinted layers onto preformed metallic nanostructures, and configurations in which the plasmonic substrate and recognition layer are prepared separately and subsequently assembled [13,163]. More specific arrangements include core–shell, planar, and sandwich configurations, which differ in the spatial organization of the recognition and plasmonic components [163,264]. In addition to the physical architecture, different imprinting strategies, such as the use of dummy templates, multifunctional monomers, and stimuli-responsive polymers, have been explored to improve recognition properties and analytical performance [13]. Surface molecular imprinting has also emerged as a promising approach to improve the accessibility of recognition sites and facilitate analyte transport toward the SERS-active regions [262]. These design strategies determine the spatial proximity among the analyte, the MIP recognition sites, and the SERS-active regions, thereby influencing both molecular recognition and Raman signal enhancement.

Beyond integrated MIP–SERS sensing platforms, molecularly imprinted materials have also been employed as selective sorbents for analyte extraction and enrichment prior to SERS measurements. In these configurations, the MIP serves as a sample preparation material rather than as an integral component of the sensing interface. The extracted analyte is subsequently analyzed using an independent SERS-active substrate. Although this approach can improve selectivity and reduce matrix interference, it represents a complementary analytical strategy rather than an integrated MIP–SERS sensor [265].

Representative studies illustrate the versatility of integrated MIP–SERS platforms across different analytical applications. An MIP–SERS platform based on silver nanoparticles was developed for melamine detection in tap water and milk, combining selective recognition by the MIP with the enhanced Raman response of the plasmonic substrate [266]. More recently, an AgNPs@MIP platform prepared by precipitation polymerization and in situ reduction of silver ions was reported for malathion detection. The sensor exhibited a limit of detection of 0.005 µg/mL and was evaluated in spiked drinking and tap water samples, with recoveries ranging from 93.0% to 100.5% [267]. In addition, recent developments have explored the application of MIP–SERS platforms for cancer biomarker detection, extending their potential use toward bioanalytical and diagnostic applications [264].

Complementary approaches involving MIP-assisted sample preparation have also been reported for the determination of pesticides and pharmaceutical compounds in food and biological samples. Examples include the selective extraction of bitertanol from fruit and vegetable samples using MIPs, followed by SERS detection [265], and the extraction of cloxacillin from porcine serum using magnetic MIPs, followed by analysis with an independent SERS substrate [268]. These approaches illustrate the potential of combining selective molecular extraction with SERS detection for trace analysis in complex matrices.

Despite these advances, several challenges remain for the practical implementation of MIP–SERS systems. A major limitation is the reproducibility of the plasmonic substrate. Differences in nanoparticle size, interparticle distance, surface coverage, and hot-spot distribution can produce substantial variations in Raman enhancement between substrates [13]. Variations in the thickness and spatial distribution of the MIP layer can introduce additional sources of signal variability. Furthermore, matrix interference, fabrication costs, scalability, and the lack of standardized procedures for substrate preparation and performance evaluation remain important barriers to practical applications, particularly in complex biological samples [163]. Reliable MIP–SERS systems therefore require careful control of both the recognition layer and the plasmonic interface, together with further improvements in fabrication reproducibility and analytical validation.

#### 5.2.3. Surface Plasmon Resonance (SPR)-Based MIP Sensors

Surface plasmon resonance (SPR) is an optical transduction technique that enables the real-time monitoring of molecular interactions at metal–dielectric interfaces. In conventional SPR configurations, polarized light is directed onto a thin metallic film, typically gold, under conditions that generate surface plasmon resonance [269]. The binding of target molecules to an MIP layer immobilized on the metallic surface changes the local refractive index, which can be monitored through shifts in the resonance angle or wavelength, or variations in the reflected light intensity, depending on the SPR configuration. By combining the molecular recognition capability of MIPs with the sensitivity of SPR to changes in the interfacial refractive index, these platforms enable label-free detection of chemical and biological analytes [1].

Different strategies have been explored to integrate MIPs into SPR sensing interfaces, depending on the polymer architecture and the desired recognition properties. One approach involves the direct formation of thin imprinted polymer films on metallic substrates, typically through surface-initiated or in situ polymerization. This strategy provides direct contact between the recognition layer and the plasmonic interface, as demonstrated by an SPR sensor developed for profenofos detection using an ultrathin MIP film polymerized directly onto a gold chip [270]. Alternatively, pre-synthesized MIP nanoparticles (nanoMIPs) can be immobilized onto gold-coated SPR chips. This approach allows the polymer particles to be prepared and characterized separately before their integration into the optical transducer. For example, Sari et al. developed an SPR nanosensor for erythromycin detection by immobilizing molecularly imprinted nanoparticles, previously synthesized by miniemulsion polymerization, onto a gold surface [271].

Another strategy involves incorporating metallic nanoparticles, particularly gold nanoparticles (AuNPs), into the MIP matrix to modify the optical response of the sensing interface. In these systems, the interaction between embedded nanoparticles and the underlying gold film can influence the local electromagnetic field and the SPR response. Matsui et al. demonstrated this approach in a dopamine sensor, in which analyte-induced swelling of the imprinted polymer increased the separation between embedded AuNPs and the gold substrate, resulting in a measurable shift in the SPR angle [272]. A related strategy was subsequently applied to atrazine detection in acetonitrile, where the coupling effect between AuNPs and the gold film enhanced the sensor response, enabling detection at concentrations as low as 5 pM [273].

MIP-based SPR sensors have been investigated for the detection of different classes of analytes, including environmental contaminants, food-related compounds, pharmaceuticals, and biomolecules [1,274]. Their main advantages include label-free operation, real-time monitoring of binding interactions, and the possibility of integrating selective recognition layers with compact optical transducers [1]. Nevertheless, their analytical performance depends on the thickness, uniformity, and stability of the MIP layer, as well as on the accessibility of the imprinted sites and the magnitude of the refractive-index change produced by analyte binding [1]. Detection of small molecules can be particularly challenging because their binding may generate relatively small changes in the local refractive index [274]. In addition, non-specific adsorption and variations in sample composition, temperature, and bulk refractive index may affect the sensor response [274]. Consequently, careful control of the sensing interface and appropriate reference measurements is essential for achieving reliable and reproducible analytical performance.

## 6. Perspectives and Challenges

The development of MIP-based sensors has resulted in a wide range of recognition materials, architectures, and transduction strategies. However, improvements in material design and analytical figures of merit do not necessarily translate into reliable performance in practical applications. The challenges that remain are therefore related not only to the molecular recognition properties of MIPs, but also to analytical validation, reproducibility, integration with real samples, and the requirements of practical sensor operation. The following sections discuss these aspects and the main factors that currently limit the transition of MIP-based sensors from proof-of-concept studies to more robust analytical applications.

### 6.1. From Proof-of-Concept to Analytical Validation

Many MIP-based sensor studies still focus on demonstrating analytical performance under controlled laboratory conditions, often using standard solutions and emphasizing parameters such as linear range, sensitivity, and limit of detection (LOD). These parameters are important for characterizing a sensor, but a low LOD does not necessarily indicate that the sensor can provide reliable results in practical applications. In real samples, molecular selectivity, matrix effects, precision, stability, and reproducibility can affect the analytical response and, consequently, the accuracy of the measurement [18,28].

The contribution of molecular imprinting should first be evaluated by comparing the MIP with a non-imprinted polymer (NIP) prepared under comparable conditions. The NIP provides information about non-specific interactions and helps to distinguish the response associated with the imprinted sites from contributions arising from the polymer matrix, electrode surface, or incorporated nanomaterials [12]. The use of structurally related compounds and relevant interferents is also necessary to determine whether the observed selectivity results from the imprinted sites or from other interactions occurring at the sensing interface [275]. This distinction becomes particularly important in hybrid sensors, in which the electrode and additional nanomaterials can contribute substantially to the measured response.

The behavior of the sensor in the sample matrix should also be considered. Recovery experiments using spiked samples are useful for evaluating the effect of the matrix on the analytical response, but satisfactory recovery does not necessarily demonstrate accurate determination of the analyte in an authentic sample. Whenever possible, the sensor should therefore be evaluated using representative real samples and, when appropriate, matrix-matched calibration or another strategy to compensate for matrix effects. Comparison with an established analytical method or reference measurement provides additional evidence of the accuracy of the proposed sensor. This aspect is particularly relevant for food, environmental, clinical, and forensic samples, in which proteins, lipids, salts, pigments, particulate matter, and other components can affect analyte transport, molecular recognition, and signal generation [276].

Reproducibility is another important aspect that is often not adequately distinguished from repeatability. Replicate measurements performed using the same sensor provide information about repeatability, but do not demonstrate that independently prepared sensors will provide the same response. In MIP-based sensors, differences in polymerization conditions, film thickness, template removal, electrode surface, nanomaterial loading, and recognition-site distribution can contribute to variations between sensors. For this reason, sensor-to-sensor and, particularly, batch-to-batch reproducibility should be evaluated whenever possible. Storage stability and operational stability should also be distinguished. A sensor may maintain its response during successive measurements over a short period while undergoing changes during storage or prolonged contact with the sample. These factors can become important when the same fabrication procedure is transferred to different electrode batches or laboratories [277].

Template removal can also affect the reliability of the analytical response. Incomplete removal leaves part of the template associated with the polymer and can reduce the accessibility of the recognition sites. Residual template can also be released during subsequent measurements and produce an additional response, a phenomenon commonly referred to as template bleeding. This effect is particularly relevant in trace analysis, when the concentration of the residual template may become comparable to the concentration of the analyte being measured. Dummy-template strategies and alternative template removal procedures can reduce this problem, but their effectiveness needs to be demonstrated experimentally [278,279].

For reusable sensors, the stability of the recognition layer should also be evaluated after successive measurement and regeneration cycles. The number of cycles, regeneration conditions, recovery of the initial response, and possible changes in selectivity or memory effects provide information about whether the MIP maintains its recognition properties during repeated use [280]. For disposable sensors, regeneration is not a relevant requirement, but reproducibility during fabrication and the variability between individual devices become more important [277]. Thus, the criteria used to evaluate a sensor should be related to its intended mode of operation rather than applied uniformly to all MIP-based devices.

Finally, the analytical performance of an MIP sensor should be considered in relation to the complete measurement procedure. Response time, sample preparation, number of operational steps, reagent and sample consumption, calibration requirements, and cost can be as relevant as the LOD when evaluating the practical application of the sensor. A very low LOD does not necessarily represent an analytical advantage if the measurement requires extensive sample treatment, several washing steps, long equilibration times, or frequent recalibration. Conversely, a sensor with a higher LOD may still be adequate for its intended application if it provides accurate results in real samples with limited pretreatment, short analysis time, and reproducible operation. The evaluation of MIP-based sensors should therefore consider whether the complete analytical procedure is appropriate for the intended application, rather than relying on analytical sensitivity as the main indicator of performance [281].

### 6.2. MIPs for Biomacromolecules: Epitope Imprinting and NanoMIPs

The recognition of biomacromolecules remains one of the more challenging applications of molecular imprinting because proteins and other large biological targets have complex structures that are strongly affected by their surrounding environment. Their size, conformational flexibility, and sensitivity to pH, temperature, and solvent composition can affect template–monomer interactions and the formation of the recognition sites. In addition, the use of non-physiological conditions during polymerization can modify the conformation of the template and, consequently, the structure of the resulting binding sites [282,283].

Epitope imprinting provides an alternative to the use of the entire biomolecule as template. In this approach, a peptide sequence derived from the target protein is used to generate recognition sites. The smaller template facilitates polymerization and template removal and allows the peptide sequence to be synthesized and chemically modified before imprinting [284]. This characteristic is particularly relevant when the target protein is difficult to obtain in sufficient purity or when systematic modification of the template is required. However, the use of a short peptide does not necessarily reproduce the structure of the corresponding region in the native protein. The selected epitope should therefore be accessible in the target molecule and provide sufficient structural discrimination from other proteins [285].

Epitope selection is consequently one of the critical steps in this approach. Sequence uniqueness alone may not be sufficient to obtain selective recognition, particularly when short peptide sequences are used. The accessibility of the epitope on the native protein, its local conformation, the presence of post-translational modifications, and possible similarities with other proteins should also be considered [282,286]. An epitope that is well represented in the peptide template but poorly exposed to the native protein may result in recognition sites that are difficult to access. Conversely, a structurally exposed and sufficiently discriminating sequence may provide a more appropriate template for recognition of the intact biomolecule [286].

The use of nanoMIPs provides another approach to improve the recognition of large targets. Because a greater fraction of the recognition sites can be located at or near the particle surface, nanoMIPs can reduce the diffusion limitations associated with deeply buried sites in conventional MIPs. This configuration is particularly relevant for proteins and other biomacromolecules, for which steric limitations can restrict access to recognition sites within highly crosslinked polymer networks [159].

The combination of nanoMIPs with solid-phase imprinting has further increased the control that can be obtained during the preparation of these materials. Immobilization of the template on a solid support changes the way in which the target is presented during polymerization and can also facilitate template recovery and the separation of nanoparticles according to their interaction with the immobilized template [94]. However, the solid support is not simply an inert substrate. Template density, orientation, surface chemistry, and polymer growth at the support can affect the composition and binding properties of the resulting nanoparticles. Recent results obtained using different solid supports demonstrated differences in particle size, yield, polymer composition, and binding affinity even when the initial monomer formulation was kept constant [158]. This observation indicates that solid-phase imprinting does not eliminate the variability associated with MIP preparation but introduces additional parameters that need to be controlled.

These aspects become particularly important when nanoMIPs are intended for biomarker detection. A recent example combined epitope imprinting, solid-phase synthesis, molecularly imprinted nanogels, and electrografting onto screen-printed electrodes for the determination of cardiac troponin T. The sensor showed a linear response from 0.01 to 0.3 ng mL^−1^, a detection limit of 0.004 ng mL^−1^, low response to interfering proteins, and detection in undiluted serum [162]. The results demonstrate the feasibility of combining different levels of control, from template selection to nanoscale recognition and electrode integration, in a single sensing platform. However, the performance of a single sensor should not be considered sufficient to establish the general superiority of nanoMIPs or epitope-imprinted materials over conventional MIPs or biological receptors. Reproducibility of the recognition sites, stability in biological matrices, control of particle populations, and performance using independent samples remain important aspects for further evaluation.

The development of MIPs for biomacromolecules will therefore depend not only on improving polymerization strategies, but also on obtaining better control over the relationship between the template structure and the recognition sites formed in the polymer. In this context, computational approaches can assist in the selection of peptide sequences by considering factors such as surface exposure, structural uniqueness, and interactions with functional monomers before experimental synthesis. The combination of rational epitope selection, solid-phase nanoMIP synthesis, and suitable transducers may provide a more controlled approach for the development of synthetic receptors for proteins and other biomacromolecules [287].

### 6.3. Computationally Guided MIP Design

The selection of functional monomers and other components of MIP synthesis has traditionally been based mainly on experimental optimization. Different monomers, template-to-monomer ratios, solvents, and polymerization conditions are tested until a material with suitable recognition properties is obtained. Although this approach can be effective, it requires a relatively large number of experiments and does not always provide information about the interactions responsible for the observed recognition. Computational methods can be used as a complementary approach to evaluate template–monomer interactions before experimental synthesis and to assist in the selection of the components used to prepare the MIP [288,289].

Density functional theory (DFT) has been used mainly to compare the stability of complexes formed between the template and different functional monomers. Calculated interaction or binding energies can provide an indication of which monomers are more likely to interact with the target. Molecular docking can be used to evaluate possible arrangements of the template and monomer, while molecular dynamics (MD) simulations can provide information about the movement of the molecules and their interactions over time. These approaches can therefore provide different types of information about the pre-polymerization system and can be used to complement experimental optimization [289,290].

Some studies have shown that this type of computational screening can be useful for selecting functional monomers before MIP synthesis. For example, DFT calculations were used to select a functional monomer for a bisphenol A-imprinted potentiometric sensor. The resulting sensor showed improved selectivity and a lower detection limit compared with a sensor prepared using methacrylic acid [71]. A similar approach was used for the development of a sulfadiazine-imprinted potentiometric sensor, in which DFT calculations were performed to assist in the selection of the functional monomer before synthesis [291]. More recently, DFT and electrostatic potential analysis were used to select (3-aminopropyl)triethoxysilane for an oxytetracycline-imprinted optical sensing system containing a MOF [292]. These examples indicate that computational analyses can reduce the number of experimental combinations that need to be investigated, particularly during the initial selection of functional monomers.

However, the interaction calculated for a template–monomer complex does not necessarily represent the behavior of the final MIP. Calculations are generally performed using simplified molecular systems, whereas the polymer obtained experimentally is a three-dimensional and heterogeneous network. Solvent, crosslinking, polymerization conditions, diffusion, accessibility of the recognition sites, and non-specific interactions can all modify the interactions observed after polymer formation. Therefore, a monomer that exhibits a favorable interaction with the template in a computational model may not necessarily produce the MIP with the highest affinity or selectivity [292]. Computational results should therefore be considered as a guide for experimental design rather than as a direct prediction of the analytical performance of the final sensor.

Molecular dynamics can provide additional information because it considers the movement of the molecules during the simulation. Compared with calculations based on a single optimized template–monomer structure, MD can be used to evaluate the interaction of several components of the pre-polymerization mixture and to investigate the effect of solvent and molecular mobility. More recent approaches have therefore attempted to include a larger number of components of the experimental formulation in the simulations [293]. However, these calculations require greater computational resources, and their results depend on the model used, including the force field, simulation conditions, and representation of the system. For this reason, increasing the complexity of the simulation does not necessarily mean that the experimental behavior of the MIP will be predicted more accurately.

Machine learning and artificial intelligence have also been proposed as complementary tools for MIP development. In these approaches, experimental data and molecular descriptors can be used to identify relationships between the composition of the MIP and its recognition or sensing performance. Unlike DFT or MD, which are based on molecular models, machine-learning methods can use experimental results directly to identify patterns that may not be easily described by a single molecular interaction. However, the application of these methods to MIPs is still limited by the amount and consistency of the available data. Differences in templates, monomers, polymerization methods, solvents, and analytical conditions can make it difficult to combine results from different studies. Models developed using a limited dataset may therefore have restricted applicability to new chemical systems, making experimental validation essential [294].

The use of computational methods in MIP development is therefore unlikely to eliminate experimental optimization completely. Their main contribution may instead be to reduce the number of experimental conditions that need to be investigated and to provide information about the interactions involved in the formation of the recognition sites. This approach may be particularly useful for biomolecular imprinting, where computational methods can assist in the selection of exposed and structurally distinctive epitopes before experimental synthesis. The combination of computational screening, systematic experimental studies, and analytical validation can consequently provide a more rational approach to the development of MIPs and help to improve the reproducibility of the resulting recognition materials.

### 6.4. Toward Greener and More Scalable MIP Synthesis

The increasing number of MIP-based sensors reported in the literature has been accompanied by growing interest in reducing the environmental impact of their preparation. Conventional MIP synthesis can involve organic solvents, initiators, crosslinkers, and repeated washing steps for template removal and purification. Depending on the preparation method, additional operations such as grinding, sieving, centrifugation, and drying may also be required. These steps increase solvent and energy consumption and generate additional waste, particularly when the polymer requires extensive processing before it can be incorporated into a sensor [295,296]. Therefore, the environmental performance of an MIP cannot be evaluated from the polymerization step alone but should be considered together with the subsequent processing required to obtain the final material.

The use of water or other less hazardous solvents is one of the most direct approaches to reducing the use of conventional organic solvents. Aqueous polymerization can be advantageous for MIPs intended for applications in aqueous samples and can reduce some of the hazards associated with organic solvents [296]. However, the replacement of an organic solvent by water does not necessarily result in a greener or better-performing MIP. Water can interfere with hydrogen bonding and other interactions involved in template–monomer association and can consequently affect the formation of the pre-polymerization complex and the structure of the resulting polymer [295]. In addition, the amount of water used, as well as the energy and resources required for washing, purification, and drying, should also be considered. Thus, solvent selection needs to be evaluated together with its effect on both imprinting efficiency and the subsequent processing of the material.

A different approach is provided by solvent-free or low-solvent processes, including mechanochemical synthesis. In mechanochemistry, mechanical energy generated by grinding or milling is used to promote chemical transformations, reducing or eliminating the need for a conventional bulk solvent phase. Its application to molecular imprinting is particularly interesting because template, monomer, and other components can be processed under solid-state or liquid-assisted grinding conditions. A solvent-free mechanochemical MIP synthesis has been reported to produce a material with template rebinding comparable to that of a conventionally prepared MIP, together with improved chemoselectivity for the investigated system [297]. However, the reduction or elimination of solvent does not by itself establish that the process is more sustainable. Milling energy, processing time, formulation, reproducibility, yield, and the purification required after synthesis also need to be considered. Mechanochemical imprinting is therefore an interesting alternative, but its environmental and scale-up advantages still need to be demonstrated for different MIP systems [298].

Template removal is another important source of solvent consumption and waste. Depending on the MIP and template, extraction can require repeated washing, large volumes of solvent, Soxhlet extraction, centrifugation, or other separation procedures [296]. This step can become particularly demanding when the template is strongly retained within the polymer or when complete removal requires several extraction cycles. Surface-imprinted and solid-phase approaches can facilitate template removal because a larger fraction of the template is located near an accessible surface, while immobilized templates can potentially be recovered and reused [94]. Template recovery is particularly relevant when expensive or difficult-to-obtain molecules are used. Recent work has also investigated the recovery of templates from MIP washing solutions, indicating that template recycling can be incorporated into the preparation process rather than considered only as a waste management step [299].

Scale-up introduces additional challenges that are not necessarily evident during laboratory-scale synthesis. Increasing the reaction volume can change mixing, heat and mass transfer, local concentrations of monomers and initiators, and polymerization kinetics. These changes may affect particle formation, morphology, and recognition-site distribution and can consequently result in differences between batches prepared at different scales [300]. This issue is particularly relevant for MIP nanoparticles and thin films, for which variations in particle size, surface chemistry, functionalization, or film thickness can affect the analytical response of the sensor. Therefore, the development of scalable MIP synthesis requires control of the variables that determine polymer formation rather than simply increasing the amounts of reagents used in a laboratory protocol [300].

The evaluation of whether an MIP synthesis is environmentally favorable should therefore consider the complete preparation procedure. The type and number of monomers and crosslinkers, solvent consumption, energy required for polymerization and processing, template removal, purification, waste generation, and template recovery can all contribute to the environmental impact of the material. The AGREEMIP framework was developed to provide a more comprehensive evaluation of these parameters rather than considering a single characteristic of the synthesis [301]. This type of analysis is particularly relevant for MIP-based sensors because the preparation of the recognition material represents only one part of the complete analytical device. Electrode fabrication, surface modification, immobilization of the MIP, and the reagents and sample required during measurements can also contribute to the overall environmental impact [302].

Greener MIP synthesis should therefore not be associated with a single modification, such as replacing an organic solvent with water. The more relevant objective is to reduce the material and energy requirements of the complete preparation while maintaining the recognition properties required for the intended application. Approaches combining low-solvent or solvent-free processing, efficient template removal, template recovery, reduced purification, and reproducible manufacturing may provide more meaningful improvements than changes restricted to a single component of the synthesis. Future studies should consequently evaluate environmental aspects together with polymer yield, recognition properties, reproducibility, energy and solvent consumption, waste generation, and the feasibility of scale-up. This broader evaluation can help determine whether the proposed MIP preparation process is not only analytically effective but also suitable for the development of more sustainable sensing platforms [301].

### 6.5. Portable, Microfluidic, and Wearable MIP-Based Sensors

**Figure 10:**
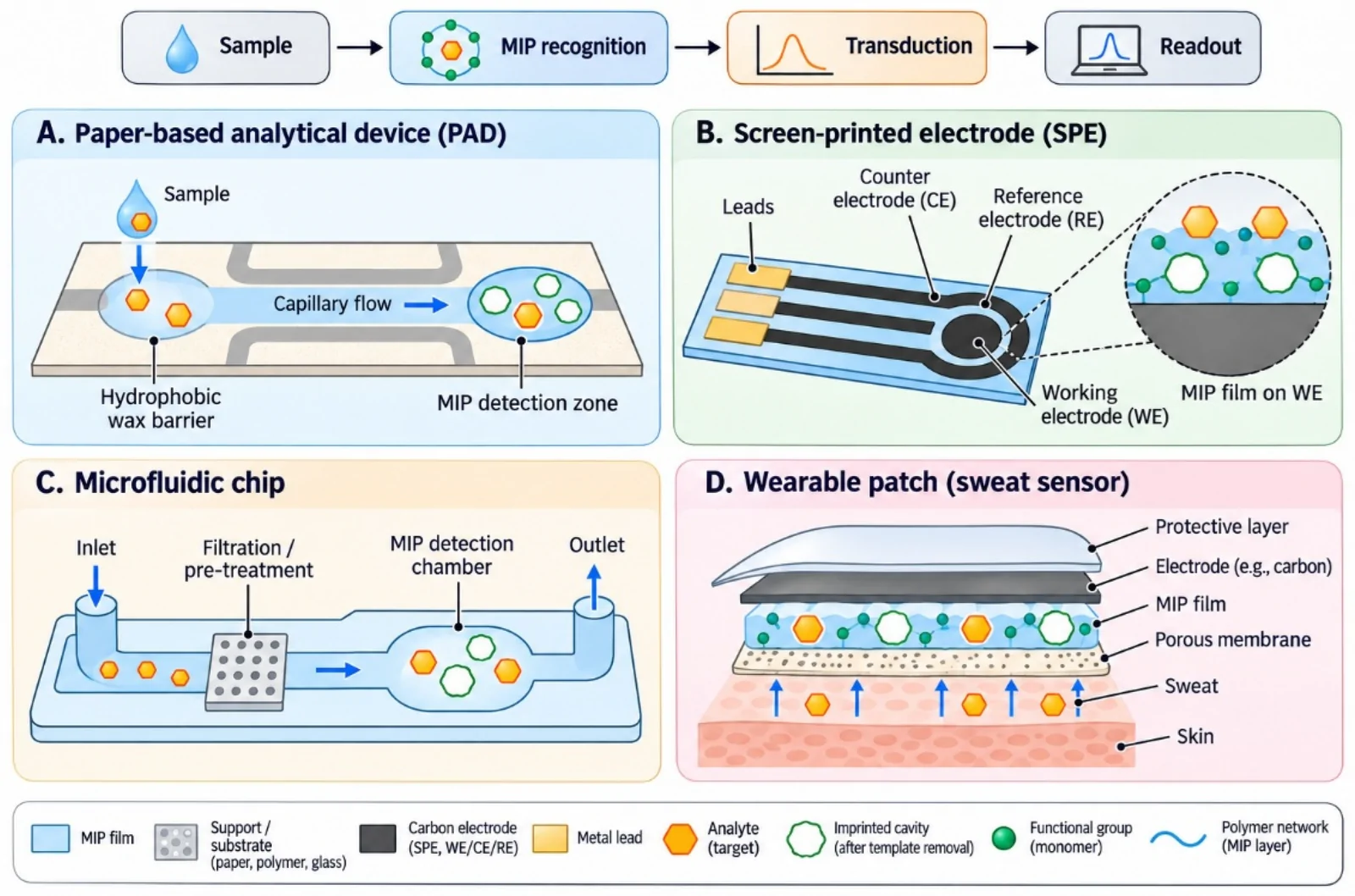
A schematic representation of portable and miniaturized MIP-based sensing platforms: (**A**) paper-based analytical devices (PADs); (**B**) screen-printed electrodes (SPEs); (**C**) microfluidic chips; and (**D**) wearable patches for sweat analysis.

The development of portable and miniaturized platforms provides a possible route for transferring MIP-based sensors from laboratory-scale systems to measurements performed closer to the sample source. Paper-based devices, screen-printed electrodes, microfluidic systems, and wearable platforms can reduce sample and reagent consumption and, depending on their configuration, allow measurements to be performed outside conventional laboratories [303,304]. However, reducing the size of the sensor does not necessarily simplify the analytical system. Miniaturization introduces additional factors related to sample transport, fabrication reproducibility, mechanical stability, and the integration of recognition and transduction components. Representative configurations of portable and miniaturized MIP-based sensing platforms, including paper-based devices, screen-printed electrodes, microfluidic systems, and wearable sensors, are illustrated in Figure 10.

Paper-based analytical devices (PADs) use the porous structure and capillary transport of paper to move the sample toward the detection region [303,304,305,306]. The characteristics of the paper, including porosity, thickness, retention capacity, and surface properties, can affect both fluid transport and the formation of the sensing interface. Hydrophilic and hydrophobic regions are therefore defined to control sample confinement and direct the solution toward the sensing area [307,308,309,310,311,312]. Wax printing, inkjet printing, laser treatment, and other patterning methods can be used for this purpose. In MIP-based devices, these characteristics are particularly relevant because the amount of sample reaching the recognition layer and the time available for interaction with the MIP are determined, at least in part, by the fluidic behavior of the paper substrate.

MIPs can be incorporated into paper-based platforms by depositing pre-synthesized polymers, electropolymerizing the recognition layer directly on paper-based electrodes, or incorporating the MIP into conductive inks [313]. These approaches differ in the location and organization of the recognition material and consequently in its interaction with the electrode and the sample. A paper-based MIP sensor for 2,4-dinitrophenol in dietary supplements, for example, showed recoveries above 85% with relative standard deviations below 10%, while electropolymerized MIPs on paper-based electrodes have been applied to hydrochlorothiazide and hydroxymethylfurfural in environmental and food-related samples [314,315,316]. These results demonstrate the feasibility of combining MIP recognition with low-cost substrates, but the analytical response depends on the reproducibility of both the recognition layer and the paper-based fluidic interface.

Screen-printed electrodes (SPEs) provide another format for reducing the size and cost of electrochemical MIP sensors. Their planar configuration and suitability for mass production facilitate the fabrication of disposable devices, while direct electropolymerization on the working electrode can reduce the number of steps required to assemble the recognition interface [317]. MIP-based SPEs have been reported for pharmaceutical and environmental analytes, demonstrating that molecular recognition can be integrated into compact electrochemical platforms [316,318]. However, the use of SPEs also introduces sources of variability associated with printed material, electrode dimensions, surface morphology, and batch-to-batch fabrication. These factors can affect the deposition of the MIP film and, consequently, the electrochemical response [317,319].

Microfluidic systems introduce an additional level of integration because sample transport and, in some cases, sample preparation can be performed within the same device used for detection. The small dimensions of microfluidic channels reduce the distance between the sample and the sensing interface and allow for the use of small sample volumes. In MIP-based systems, this configuration can also be used to control analyte delivery, perform sample pretreatment, and operate under continuous-flow conditions [320]. A capillary microfluidic MIP sensor, for example, combined plasma separation and electrochemical detection of metabolites directly from whole blood, reducing the need for conventional sample pretreatment [321]. Such systems illustrate that miniaturization can involve more than reducing the dimensions of the transducer: sample handling and molecular recognition can also be incorporated into the same analytical platform.

Wearable MIP-based sensors extend this integration to measurements performed directly on or close to the body. Sweat is one of the most investigated matrices because it can be sampled over extended periods and contains several compounds of analytical interest. MIP-based wearable platforms have been reported for cortisol and other biomarkers using flexible electrodes, sweat-transport membranes, and integrated microfluidic components [322,323]. A wearable MIP electrochemical sensor combining a flexible gold electrode with a directional sweat-transport membrane was used for cortisol measurements over repeated regeneration cycles [324]. Other systems have combined MIP-based electrochemical detection with wearable microfluidics for the analysis of cortisol and glucose in human sweat [325]. In these systems, however, the MIP represents only one part of the analytical process. Sweat collection, transport to the recognition layer, changes in sample composition, mechanical deformation, electrode stability, and signal acquisition can all affect the measured response.

An important distinction should be made between repeated measurements, in situ analysis, and continuous monitoring in wearable MIP-based sensors. Repeated measurements involve successive determinations separated by incubation, washing, or regeneration steps, whereas in situ analysis refers to measurements performed directly at the sampling site and does not necessarily imply uninterrupted monitoring [326]. Genuine continuous monitoring requires the sensor to follow temporal changes in analyte concentration during operation, ideally under dynamic conditions involving both increasing and decreasing concentrations [326]. This distinction is illustrated by wearable MIP systems that have demonstrated repeated cortisol measurements over multiple regeneration cycles but require an extraction step between determinations [327]. Other platforms have demonstrated real-time monitoring through the integration of MIP recognition with paper-based microfluidics, although their evaluation of dynamic responses does not necessarily establish reversible tracking of changing analyte concentrations [327]. In contrast, wearable platforms integrating MIP recognition with in situ electrochemical regeneration, microfluidic sweat sampling, and signal processing have demonstrated more advanced configurations for prolonged cortisol monitoring [203,328]. These approaches should nevertheless be assessed according to their ability to track both increasing and decreasing analyte concentrations, rather than solely by the number of measurements performed or the duration of device operation. In addition, the absence of a manual regeneration step does not necessarily establish regeneration-free operation, particularly when an automated or electrochemically induced regeneration process is incorporated into the device [203,328]. Therefore, studies should explicitly report whether regeneration is required, how it is performed, and whether the analytical response can be restored reproducibly over successive cycles [203,324,328].

Despite these advances, the available evidence remains insufficient to establish reliable reversible tracking of both increasing and decreasing analyte concentrations under controlled dynamic conditions across the reported wearable MIP platforms. Repeated measurements after regeneration and the observation of physiological concentration profiles demonstrate important progress, but do not necessarily confirm the ability of a sensor to follow successive concentration increases and decreases without hysteresis, carryover, or baseline drift. Further studies should therefore include controlled concentration-step experiments in both directions, together with the evaluation of signal recovery, response and recovery times, and baseline stability.

The operational stability of wearable MIP sensors should also be evaluated beyond their initial analytical performance. Baseline drift, changes in signal amplitude, and incomplete recovery after analyte removal may compromise the interpretation of concentration changes during prolonged measurements [326]. A sensor may retain an acceptable response over repeated cycles while exhibiting progressive changes in its baseline or sensitivity. Consequently, cycle-to-cycle reproducibility alone is insufficient to establish long-term monitoring capability. This distinction is particularly relevant when comparing repeated measurements with continuous operation, since operational stability should be assessed under prolonged exposure to a constant analyte concentration, whereas storage stability is evaluated through measurements performed at different time points [326]. Studies on wearable MIP sensors have reported reproducible responses over multiple regeneration cycles and retention of the analytical signal during storage, although these results do not necessarily establish baseline stability during uninterrupted operation [327]. Relevant evaluations should therefore include baseline stability during operation, signal recovery after regeneration, residual analyte carryover, and the ability to distinguish genuine changes in analyte concentration from changes associated with the sensing interface or the transducer [326]. The distinction between analytical stability during repeated measurements and stability during uninterrupted or prolonged operation is therefore essential when comparing wearable MIP platforms.

The performance of wearable MIP-based sensors may also be affected by variations in physiological and environmental conditions, particularly temperature and pH. Changes in skin and sweat temperature during physical activity or exposure to different environmental conditions may influence analyte diffusion, binding equilibria, polymer swelling, and electrochemical reaction kinetics, potentially affecting the measured response [329,330]. Nevertheless, systematic evaluations of the thermal stability of wearable MIP recognition layers under realistic variations in skin and sweat temperature remain limited.

Similarly, sweat pH can vary according to physiological conditions, sampling location, and sweat secretion rate. In particular, changes in sweat flow rate affect bicarbonate reabsorption in the sweat ducts, resulting in variations in the pH of the collected sweat [331,332]. These variations may alter the ionization state of both the target analyte and the functional groups within the MIP, thereby affecting molecular recognition and signal transduction. In pH-responsive MIPs, changes in the ionization of functional groups can also modify polymer conformation and swelling behavior, potentially influencing analyte binding and release [333]. Such effects are particularly relevant when measurements are performed directly in unbuffered sweat rather than in controlled laboratory solutions.

Strategies to address these limitations include incorporating temperature and pH sensors into the wearable platform, applying appropriate signal compensation, or introducing in situ buffering through integrated microfluidic components [199,329]. Although buffering can help maintain more controlled conditions for molecular recognition, its implementation should consider possible sample dilution, changes in ionic strength, and the consumption or depletion of buffering reagents, which may themselves influence the analytical response. Moreover, the effect of the buffering strategy on analyte recovery and sample transport should be considered. Therefore, simultaneous monitoring of relevant matrix parameters or appropriate matrix correction may be necessary to ensure reliable measurements under variable physiological conditions [199,329].

The evaluation of wearable MIP sensors should also distinguish analytical performance in different sample matrices. Measurements in buffer or artificial sweat are useful for initial characterization under controlled conditions, including the assessment of selected interferents and matrix components [327,334]. However, they do not fully reproduce the variability of collected human sweat or the dynamic conditions encountered during on-body operation. Studies using wearable MIP platforms have demonstrated analysis of collected human sweat [335] and on-body measurements in volunteers [327,334,336]. These validation levels provide complementary rather than interchangeable evidence. For example, a sensor calibrated in artificial sweat should not be considered validated for on-body monitoring without further assessment in human samples and under the intended operating conditions. Whenever possible, on-body results should be compared with an independent analytical method or with independently collected sweat samples to establish the reliability of the wearable measurement [334].

Finally, the time required to obtain an analytical result should be evaluated at the level of the complete wearable platform rather than being restricted to the intrinsic response time of the MIP recognition layer. The intrinsic response time describes the time required for analyte–MIP interactions and the associated signal transduction to produce a measurable response under defined experimental conditions. Wearable MIP studies have reported response times ranging from less than 2 min to approximately 3 min, depending on the sensor architecture and experimental conditions [327,335]. However, this parameter does not account for delays introduced by the other components of the device. In practical wearable applications, the total measurement time may also include sweat induction, sample collection, transport through microfluidic channels, filling of the sensing chamber, signal stabilization, and data acquisition [326,337]. These additional steps may introduce delays between the physiological event and the corresponding analytical result, even when the MIP itself exhibits a relatively rapid response. This distinction is particularly important for continuous monitoring, in which the effective temporal resolution depends not only on the sensor response time but also on sample renewal, fluid transport, and the time required for surface regeneration. For example, a wearable MIP platform integrating paper-based microfluidics required approximately 2 min to fill the sensing chamber and 3 min to remove the sweat sample, in addition to the time required for the sensor response to reach equilibrium [327]. Moreover, slow sample exchange or incomplete analyte removal during regeneration may result in signal delays, carryover, or reduced ability to track rapid changes in analyte concentration. Therefore, reporting only the response time of the recognition layer may overestimate the temporal performance of the complete device. A more comprehensive evaluation should distinguish sensor response and recovery times from the total measurement time and the effective sampling interval, while considering transport delays, sample renewal, regeneration requirements, and operational stability under prolonged exposure to real sweat [326,337]. These parameters are essential for determining whether a wearable MIP platform is suitable for discrete measurements, repeated analysis, or reliable continuous monitoring under dynamic physiological conditions.

The challenges associated with these platforms extend beyond the analytical performance of the MIP itself and involve the reproducible integration of multiple components operating simultaneously. Variations between fabricated devices, changes in fluid transport, mechanical deformation, long-term changes in the recognition layer, and differences in sample composition can all affect the analytical response [338,339]. These effects may become more pronounced when sample collection and pretreatment are incorporated into the device, since variations in these steps are directly transferred to the measurement [338]. Consequently, validation under the conditions in which the device is intended to operate is essential, rather than relying only on measurements performed with standard solutions [321,327].

The environmental aspects should also be considered when evaluating disposable and wearable MIP-based platforms. A paper-based or disposable sensor is not necessarily a more sustainable analytical device, since its environmental impact depends on the materials used, manufacturing process, incorporated nanomaterials, reagents, and final disposal [340,341]. Similarly, reducing sample volume does not necessarily compensate for the environmental impact associated with producing and discarding individual devices. The development of portable MIP sensors should therefore consider fabrication reproducibility, sample handling, calibration, operational stability, and disposal together with analytical performance.

### 6.6. Reproducibility, Stability, and Manufacturing Challenges

Reproducibility is an important limitation for the development of MIP-based sensors, particularly when the analytical performance obtained with a small number of laboratory-prepared devices is extrapolated to a larger number of sensors. Variability can occur at different levels, including replicate measurements using the same sensor, independently prepared sensors, different synthesis batches, and different electrode batches. In MIP-based systems, these differences can result from variations in polymerization conditions, monomer and crosslinker ratios, template removal, film thickness, surface functionalization, nanomaterial loading, and the distribution and accessibility of the recognition sites [300,342].

The electrode can introduce an additional source of variability that is independent of the MIP itself. Differences in surface roughness, electroactive area, electrode geometry, ink composition, surface pretreatment, and coating uniformity can modify charge transfer and analyte accessibility. This issue is particularly relevant for screen-printed and other mass-produced electrodes, for which small differences between substrates can affect the response of nominally identical sensors [317]. Therefore, replicate measurements performed using a single electrode provide information about measurement repeatability, whereas measurements using independently prepared sensors are needed to assess sensor-to-sensor reproducibility [300,343].

For this reason, reproducibility should be evaluated using independently prepared sensors and, whenever possible, independent synthesis batches. This distinction is particularly important for MIPs because the recognition properties depend on the conditions used during polymer formation and template removal. A similar nominal composition does not necessarily result in the same distribution of recognition sites if polymerization, deposition, or extraction conditions vary. The same consideration applies to hybrid systems, in which differences in the amount or distribution of a nanomaterial can modify the electrochemical interface independently of changes in molecular recognition [278,300].

Stability should also be evaluated according to the conditions under which the sensor is intended to be used. Storage stability indicates whether the sensor maintains its analytical response after a defined storage period, whereas operational stability refers to changes occurring during repeated measurements or prolonged operation. In reusable MIP sensors, regeneration can progressively modify the polymer network or the accessibility of the recognition sites, particularly when strong solvents, pH or ionic strength changes, or repeated electrochemical treatments are used. Temperature and prolonged contact with the sample can also affect the polymer and the electrode interface [343,344].

Template removal can also be related to stability and reproducibility. Residual template or other weakly retained compounds may be released during subsequent measurements and contribute to the analytical response, particularly in trace analysis. Incomplete extraction can also leave recognition sites partially occupied and alter the response obtained in the first measurements. Thus, the template removal procedure should be considered together with the subsequent use of the sensor, rather than only as a step performed at the end of MIP synthesis [278,345].

The difficulties associated with reproducibility become more evident when MIP preparation is transferred from laboratory-scale synthesis to larger-scale or parallel production. At laboratory scale, temperature, mixing, reaction time, electrode positioning, coating volume, and drying conditions can be controlled relatively easily. Changes in reaction volume or the number of sensors produced simultaneously can modify mass and heat transfer, reagent distribution, coating thickness, and polymerization kinetics [339]. For nanoparticle and thin-film MIPs, relatively small differences in particle size, surface chemistry, film thickness, or recognition-site accessibility can consequently result in measurable differences in sensor response. Increasing the amounts of reagents used in a laboratory protocol is therefore not sufficient to guarantee that the same material and analytical performance will be obtained on a larger scale.

Characterization and quality control can help identify the origin of these variations. Depending on the type of sensor, relevant parameters include film thickness, recognition-layer mass, surface morphology, electrochemically active area, polymer composition, particle size, and surface chemistry. For electrode-based systems, it is particularly important to distinguish changes associated with the transducer from those associated with the MIP. An increase or decrease in analytical response, for example, may result from a change in electrode area or charge transfer rather than from a change in molecular recognition [300,343]. Standardized preparation procedures and defined acceptance criteria can therefore contribute to more consistent sensor fabrication.

Process Analytical Technology (PAT) could provide an additional approach to improving the reproducibility and quality control of MIP-based sensor manufacturing. Potential applications include monitoring critical process parameters, such as polymerization temperature and reaction time, as well as critical material attributes, such as monomer conversion, film thickness, and surface properties, during material preparation and sensor fabrication [346,347,348]. Depending on the synthesis strategy, in-line, on-line, or at-line monitoring could help identify process deviations before device assembly and provide information about the relationship between manufacturing conditions and final sensor performance [347]. Combining process monitoring with predefined material and sensor acceptance criteria could improve batch-to-batch consistency and support the transition from laboratory-scale preparation to more scalable manufacturing [346]. However, the implementation of PAT in MIP-based sensor production remains an area for further development.

Another issue is the lack of uniformity in the analytical evaluation of MIP-based sensors. Parameters such as imprinting factor, selectivity, recovery, stability, and LOD are not always determined under comparable conditions. The comparison between MIP and NIP is particularly sensitive to the preparation of the non-imprinted material because changes in polymer composition, morphology, or surface properties can modify the contribution of non-specific interactions [12]. Similarly, different procedures for calculating LOD can result in values that are not directly comparable between studies. According to IUPAC terminology, the detection limit depends on the response of the blank, the statistical criterion used to distinguish the signal from the blank, and the analytical procedure adopted [349]. Consequently, the numerical value of the LOD should be interpreted together with the experimental and statistical conditions under which it was obtained.

For this reason, comparison between MIP-based sensors should not be based only on the lowest reported LOD. The number of independently prepared sensors, repeatability, sensor-to-sensor and batch-to-batch variability, calibration range, selectivity, performance in real samples, recovery, storage and operational stability, regeneration, response time, and sample preparation requirements provide additional information about the analytical performance of the system. Comparison with an established analytical method is also important when the sensor is proposed for practical applications [26,350].

The reproducibility of MIP-based sensors therefore depends on more than the molecular recognition properties of the polymer. The electrode, the polymerization procedure, the template removal step, the incorporation of additional materials, and the analytical measurement itself can all contribute to the final response. Greater control of these variables, together with characterization of independently prepared sensors and more consistent analytical evaluation, is necessary to determine whether the performance obtained in laboratory studies can be reproduced in subsequent preparations and under practical measurement conditions [18].

## 7. Conclusions

Molecularly imprinted polymers have evolved from conventional bulk recognition materials into increasingly structured systems for chemical sensing. As discussed throughout this review, the performance of an MIP-based sensor is determined not only by the chemical interactions established during imprinting, but also by the morphology, accessibility, spatial organization, and integration of the recognition layer with the transducer. Conventional preparation methods remain useful because of their simplicity and versatility, but limitations related to mass transfer, recognition-site accessibility, and morphological control have stimulated the development of surface-oriented, magnetic, hollow, nanoscale, and hybrid architectures.

The incorporation of carbon-based materials, quantum dots, metal–organic frameworks, magnetic nanoparticles, and other functional components has further expanded the possibilities for integrating molecular recognition with signal generation and transduction. These materials can influence different aspects of sensor performance, including surface area, charge or energy transfer, and optical response. In this context, electrochemical and optical platforms offer complementary approaches to MIP-based sensing, with the latter including luminescence-, SERS-, and SPR-based strategies. However, the incorporation of additional materials does not necessarily improve molecular recognition or analytical performance. Their contribution should be evaluated according to their specific function in the sensing interface, considering possible effects on recognition-site accessibility, non-specific interactions, and overall structural complexity.

Electropolymerization remains particularly relevant for electrochemical sensing because it allows for the formation of thin recognition layers directly on the electrode surface. At the same time, nanoMIPs, solid-phase imprinting, epitope imprinting, and molecularly imprinted nanogels are extending MIP technology toward more complex targets, including biomacromolecules. Computational approaches can also assist in the selection of templates and functional monomers and in the interpretation of template–monomer interactions. However, computational predictions remain dependent on experimental validation because the final recognition properties are determined within a heterogeneous polymeric environment.

Despite the large number of reported MIP-based sensors and improvements in analytical figures of merit, practical application remains dependent on factors beyond sensitivity and limit of detection. Validation in realistic matrices, appropriate NIP controls, evaluation of non-specific interactions, reproducibility between independently prepared sensors and batches, and comparison with established analytical methods are necessary to determine the actual analytical performance of these systems. Response time, regeneration efficiency, and storage and operational stability are also important for evaluating their suitability under practical conditions. Similarly, the development of portable, paper-based, microfluidic, and wearable platforms requires control of fabrication, sample handling, device stability, and the integration of recognition and transduction components. For wearable systems in particular, validation under the intended operating conditions and consideration of the total measurement time are essential.

The transition from laboratory-scale preparation to larger-scale manufacturing also requires attention to reproducibility, process control, and environmental impact. More sustainable synthesis strategies, appropriate selection and use of materials, and monitoring of critical manufacturing parameters may contribute to more consistent production and reduced environmental burden. Standardized preparation procedures, characterization of independently produced sensors, and defined quality control criteria are therefore important for assessing whether the performance obtained in laboratory studies can be maintained during larger-scale production.

Therefore, further development of MIP-based sensors should consider the complete recognition–transduction interface rather than the MIP as an isolated recognition material. Improvements in molecular design, polymer architecture, transducer integration, analytical validation, response and regeneration characteristics, reproducibility, and manufacturing control need to be considered together. This integrated approach is important for moving MIP-based sensors from laboratory proof-of-concept studies toward reproducible and analytically validated devices for practical chemical and biosensing applications.

## Figures and Tables

**Table 1:** Representative MIP-based sensors prepared by bulk polymerization, including target analytes, synthesis components, sample matrices, and transduction methods.

Analyte	Sample	Functional Monomer	Solvent	Cross-Linker	Initiator	Transducer	Response Time (s)	Linear Range (µM)	LOD (µM)	Sensitivity	Ref
Moxifloxacin hydrochloride	Human urine	MAA or 4-VP	Dimethyl sulfoxide and acetonitrile	EGDMA	AIBN	Potentiometric	≤30	10–10,000	1.7	55.9	[59]
Zileuton	Human plasma	MAA	DMSO	EGDMA	AIBN	Voltammetric	-	0.423–105.8	0.800	0.0216	[60]
Gallic acid	Edible plants	2-VP	Acetonitrile and toluene	TRIM	AIBN	Potentiometric	10	10–320	-	72.9	[61]
Benzyl methyl ketone	Aqueous samples	ItA and 1-vinylimidazole	DMF	p-divinylbenzene	AIBN	Capacitive	-	50–1000	1	0.0015	[62]
Metribuzin	Tomatoes, potatoes, and Egyscor^®^ 70% herbicide	ItA	Acetone	EGDMA	AIBN	Voltammetric	-	0.001–100	0.0005	0.1687	[63]
Chlorpyrifos	Apple, cucumber, and pomegranate	ItA	Acetonitrile	EGDMA	AIBN	Voltammetric	-	0.028–28	0.01912	4.0159	[64]
Daclatasvir	Pharmaceutical formulations, serum, and urine samples	AA or AM	DMSO	EGDMA	AIBN	Potentiometric	≤20	0.01–10,000	0.00693	30.32	[65]

AA: acrylic acid; AIBN: 2,2′-azobisisobutyronitrile; AM: acrylamide; DMF: dimethylformamide; DMSO: dimethyl sulfoxide; EGDMA: ethylene glycol dimethacrylate; ItA: itaconic acid; MAA: methacrylic acid; TRIM: trimethylolpropane triacrylate; 2-VP: 2-vinylpyridine; 4-VP: 4-vinylpyridine.

**Table 2:** Representative MIP-based sensors prepared by precipitation polymerization, including target analytes, synthesis components, sample matrices, and transduction methods.

Analyte	Sample	Functional Monomer	Solvent	Crosslinker	Initiator	Transducer	Response Time	Linear Range (µM)	LOD (µM)	Sensitivity	Ref
Creatinine	Aqueous solution of creatinine and human serum	MAA	Mixture of acetonitrile and toluene	Divinylbenzene	AIBN	Electrochemical	10 min	53–123.8	0.88	8.84	[74]
Fluoxetine (FXT)	Human blood serum (deproteinized)	ItA	Methanol	MBAA	AIBN	Voltammetric	3 min	0.5–40	0.3	0.08948	[73]
Heparin (anticoagulant)	Aqueous solutions	MAA	Methanol	EGDMA	AIBN	Potentiometric	10 s	0.1–100	1	-	[75]
p-Nitrophenol (p-NP)	Industrial effluents	MAA	Methanol	EGDMA	Benzoyl peroxide	Cyclic voltammetry	8 min	2–400	0.2	8.5835	[76]
Bisphenol A (BPA)	Environmental samples (water)	Allyl-β-CD	Acetonitrile	EGDMA	AIBN	Potentiometric	5 min	1–10	0.29	2.0	[71]

AIBN: 2,2′-azobisisobutyronitrile; CD: cyclodextrin; EGDMA: ethylene glycol dimethacrylate; ItA: itaconic acid; MAA: methacrylic acid; MBAA: N,N′-methylene-bisacrylamide.

**Table 3:** Representative magnetic MIP-based systems for sensing applications, illustrating the diversity of magnetic architectures, transduction mechanisms, and target analytes.

Nanoparticle	Transduction Mode	Analyte	Network-Forming Component	Functional Monomer	Polymerization	Response Time	Linear Range (µM)	LOD (µM)	Sensitivity	Ref
ZnFe_2_O_4_@SiO_2_-AuNCs	surface-enhanced Raman scattering (SERS)	ciprofloxacin	EGDMA	AM	Bulk	12 h	0.01–100	0.001	-	[114]
Fe_3_O_4_@SiO_2_	electrochemical/carbon paste-MIP electrode	tetracycline	EGDMA	AA	Precipitation	80 s	0.5–40	0.15	1.03 × 10^−7^ µA/dec	[115]
Fe_3_O_4_@SiO_2_-MPS	electrochemical/graphite epoxy composite electrodes	glutathione	TRIM	Acrylamide	Bulk	30 min	1–1400	0.007	0.364 µA/µM	[116]
Fe_3_O_4_/Mn-ZnS	fluorescent	chlorpyrifos	TEOS	APTES	Sol–gel	7 min	0.028–0.1526	0.00117	-	[117]
Fe_3_O_4_-MNPs/Py	colorimetric	Maleic hydrazide	EGDMA	MAA	Bulk	5 min	6.45–322.2	3.87	0.004 abs/µM	[118]
Fe_3_O_4_/Gr-UiO-66	electrochemical/SPE	cannabidiol	EGDMA	MAA	Bulk	30 min	5–100	0.05	0.049 µA/µM	[119]
Fe_3_O_4_	electrochemical/SPE	folic acid	EGDMA	MAA	Bulk	2 h	2.5–48	0.65	0.03 µA/µM	[120]
Fe_3_O_4_@SiO_2_-MPS	electrochemical/SPE	amoxicillin	MBAA	MAA	Bulk	NR	2.5–57	0.75	0.9 µA/µM	[121]
Fe_3_O_4_@SiO_2_	electrochemical/BDD	Bisphenol A	TEOS	APTES	Sol–gel	NR	5–73	0.38	0.163 µA/µM	[122]
ZnWO_4_/γ-Fe_2_O_3_	electrochemical	chloramphenicol	EGDMA	MAA	Bulk	10 min	0.12–1224	0.034	0.010 µA/µM	[123]
Fe_3_O_4_@SiO_2_	electrochemical/SPGE	ketamine	EGDMA	MAA	Bulk	4 min	1 × 10^−6^–400	8 × 10^−7^	3.89 µA/dec	[124]
Fe_3_O_4_	electrochemical/GCE	ezetimibe	EGDMA	MAA	Bulk	8 min	0.001–10	0.0007	0.4114 µA/µM	[125]

AA: acrylic acid; AM: acrylamide; APTES: 3-aminopropyltriethoxysilane; AuNCs: gold nanoclusters; BDD: boron-doped diamond; GCE: glassy carbon electrode; EGDMA: ethylene glycol dimethacrylate; LOD: limit of detection; MAA: methacrylic acid; MBAA: N,N′-methylenebisacrylamide; MNPs: magnetic nanoparticles; MPS: 3-(methacryloxy)propyltrimethoxysilane; NR: not reported; Py: pyrrole; TEOS: tetraethyl orthosilicate; SERS: surface-enhanced Raman scattering; SPE: screen-printed electrode; SPGE: screen-printed gold electrode; TRIM: trimethylolpropane triacrylate.

**Table 4:** MIP-MWCNT-based sensors, highlighting integration strategies, functionalization approaches, and their application in electrochemical sensing.

Analyte	Samples	MIP Preparation	MWCNTs Functionalization	MIP-MWCNT Integration	Response Time (s)	Linear Range (µM)	LOD (µM)	Sensitivity	Ref
Insulin	Aqueous, blood serum, and pharmaceutical formulations	Bulk	Vinyl group	Surface	15	6.8 × 10^−5^–5.682 × 10^−3^	1.86 × 10^−5^	≤0.0593	[135]
Diuron	River water	Bulk	Carboxyl	Mixture	-	0.052–1.25	0.009	0.51	[136]
Gallic acid	Fruit juices	Bulk	-	Mixture	-	0.12–380.0	0.047	0.0021	[137]
Chlorpromazine	Tablets and human urine	Suspension	Vinyl group	Surface	-	0.00075–0.25	0.000296	0.2472	[130]
Metribuzin	Tomatoes, potatoes, and Egyscor^®^	Bulk	-	Mixture	-	0.001–100	0.0005	0.1687	[63]

Surface: MIP is polymerized directly on MWCNTs; Mixture: MWCNTs are mixed with the synthesized MIP; (-): functionalization not performed.

**Table 5:** Representative MOF@MIP-based sensing systems, highlighting MIP integration strategies, transduction mechanisms, and target analytes.

MOF/Hybrid Architecture	MIP Integration Strategy	Target Analyte	Transduction Mode	Response Time (min)	Linear Range (μM)	LOD (μM)	Sensitivity (μM^−1^)	Ref
Eu/Tb-MOF	Surface imprinting	Perfluorooctanoic acid	Ratiometric fluorescence/smartphone-assisted	5/4	0.02–2.8/0.1–2.8	9.8 × 10^−4^/3.26 × 10^−3^	1787/0.2987	[185]
Ag-MOF@MC	Sol–gel	Hemoglobin	Electrochemical	17.5	2 × 10^−7^–1	9 × 10^−8^	14	[186]
Mg, N-CDs/Eu-MOFs	Sol–gel	Oxytetracycline	Ratiometric fluorescence/smartphone-assisted	2/2	0.0434–108.55/0.1737–108.55	1.43 × 10^−2^/5.60 × 10^−2^	0.0652/0.01345	[187]
Co-MOF/PLA nanofiber	Electropolymerization	Ciprofloxacin	Electrochemical (DPV)	2	0.5–150	0.017	0.016	[188]
Zn/La-MOF	Electropolymerization	Buprenorphine	Electrochemical	0.33	0.00855–0.107	0.00231	-	[189]
Zr-LMOF	Surface imprinting	Chloramphenicol	Fluorescence	5	0.0005–0.500	4.02 × 10^−5^	8.789	[190]
MOF/CdSe-ZnS QD@MIP	Reverse microemulsion	Pyrraline	Fluorescence	80	5–1000	3.9	0.0015	[191]
Cr-MOF@MIP	Surface imprinting	Tetracycline	Fluorescence	0.167 (10 s)	0.450–112.50	0.0720	0.0221	[192]
LMOF/MIP paper-based chip	Surface imprinting	Tetracycline	Fluorescence/paper-based	30	0.02079–62.38	0.01761	0.08675	[193]
AuNP@UiO-66-based MIP	Electropolymerization	Ofloxacin	Electrochemical	12	0.001–0.5/0.5–5	1.5 × 10^−4^	-	[194]
Cu-MOF/MXene/MIP	Electropolymerization	Hygromycin B	Electrochemical	5	0.005–5	0.00192	-	[195]

CDs: carbon dots; MC: mesoporous carbon; PLA: polylactic acid; LMOF: luminescent metal–organic framework; AuNPs: gold nanoparticles.

**Table 6:** A comparative overview of conventional MIP synthesis methods and advanced architectures according to their structural characteristics and relevance to sensing applications.

Strategy	Morphology	Site Accessibility	Mass Transfer	Scalability and Integration
Conventional synthesis methods				
Bulk	Irregular particles or monoliths	Often limited	Slow; long diffusion paths	Simple synthesis; requires immobilization
Precipitation	Uniform spherical particles	Generally improved	Faster; short diffusion paths	Potentially scalable; particle immobilization required
Emulsion	Droplet-controlled particles	Depends on particle size	Generally favorable	Requires emulsion and surfactant control
Suspension	Spherical particles	Depends on particle structure	Depends on particle size	Requires stabilizer control
Sol–gel	Porous inorganic or hybrid networks	Depends on porosity	Pore-dependent	Sensitive to synthesis conditions
Advanced architectures				
Magnetic MIPs	Magnetic core–polymer shell	Favorable in surface-oriented systems	Facilitated by particle dispersion	Magnetic separation; optical/electrochemical integration
Carbon-based MIPs	MIP–carbon composites	Depends on MIP coverage	May benefit from porous supports	Particularly suitable for electrochemical sensors
Hollow MIPs	Hollow particles with imprinted shells	Potentially high	Facilitated by hollow structures	More complex synthesis
Restricted-access MIPs	Functionalized porous structures	Selective surface accessibility	Depends on pore structure	Useful for complex matrices
NanoMIPs	Imprinted nanoparticles	Generally high	Short diffusion paths	Particle recovery and reproducibility challenges
Nanogels	Soft cross-linked networks	Depends on swelling	Governed by network structure	Requires control of crosslinking
MOF–MIP hybrids	Polymer–MOF composites	Depends on MIP coverage	Potentially enhanced by MOF porosity	Framework stability and integration challenges

**Table 9:** Representative CQD-based MIP sensors, emphasizing fluorescence-based detection and the incorporation of carbon-derived quantum dots as signal-generating components.

Nanoparticle	Sensor Type	Analyte	Solvent	Initiator	Polymerization	Response Time (min)	Linear Range (μM)	LOD (μM)	Sensitivity (μM^−1^)	Ref
N-GQDs	fluorescent	terbuthylazine	Ethanol	NH_3_ 25%	Sol–gel	20	8.71 × 10^−4^–0.44	2.61 × 10^−4^	0.042	[243]
ZnO@N-GQDs	fluorescent	Diflunisal	Methanol	NH_3_ 25%	Sol–gel	16	4.00 × 10^−4^–0.20	1.20 × 10^−4^	0.116	[244]
N,S-CQDs	fluorescent	chloramphenicol	cyclohexane, triton and butanol	NH_3_ 25%	Reverse microemulsion	3	0.24–26.1	7.65 × 10^−3^	1.075 × 10^5^	[245]
GQDs	fluorescent	Biotin	Ethanol	NH_4_OH	Sol–gel	6	0.40–6.70	0.32	0.153	[246]
melanin@N-GQDs	fluorescent	Prometryn	Ethanol	NH_3_ 25%	Sol–gel	16	0–0.41	2.65 × 10^−3^	7.7	[247]
B/GCDs and B/RCDs	fluorescent	aflatoxin B1 and zearalenone	Ethanol	NH_3_·H_2_O	Sol–gel	10	3.2 × 10^−5^–0.32 and 9.43 × 10^−5^–0.31	1.02 × 10^−5^ and 5.66 × 10^−5^	15,608 and 13,275	[101]
NCQDs/SiO_2_	fluorescent	Sudan III	n-hexane	NH_3_ 25%	Sol–gel	15	0.005–0.80	1.39 × 10^−3^	1.630	[248]
OH-HNTs@N-GQDs@Fe_3_O_4_	fluorescent	marbofloxacin	Ethanol	NH_4_OH	Sol–gel	20	2.76 × 10^−4^–0.069	8.28 × 10^−5^	0.671	[249]
N-GQDs-ZnO/CF-Fe_3_O_4_	fluorescent	Sparfloxacin	Ethanol	NH_3_ 25%	Sol–gel	18	2.55 × 10^−4^–0.26	2.55 × 10^−4^	0.004	[250]
GQDs@PC	fluorescent	sulfadimethoxine	Ethanol	NH_3_ 25%	Sol–gel	16	3.22 × 10^−4^–0.08	9.67 × 10^−5^	0.042	[251]

TEOS was used as the crosslinker and APTES as the functional monomer in all materials. APTES: 3-aminopropyltriethoxysilane; B/GCDs: blue/green carbon dots; B/RCDs: blue/red carbon dots; CF: carbon foam; CQDs: carbon quantum dots; GQDs: graphene quantum dots; HNTs: halloysite nanotubes; LOD: limit of detection; NCQDs: nitrogen-doped carbon quantum dots; N-GQDs: nitrogen-doped graphene quantum dots; N,S-CQDs: nitrogen and sulfur co-doped carbon quantum dots; PC: porous carbon; TEOS: tetraethyl orthosilicate.

**Table 10:** Representative quantum dot-based MIP sensors, including QD composition, sensing mode, target analytes, and MIP preparation conditions.

Nanoparticle/ Composite	Sensor Type	Analyte	Cross-linker	Functional Monomer	Solvent	Initiator	Polymerization	Response Time (min)	Linear Range (μM)	LOD (μM)	Sensitivity (μM^−1^)	Ref
CdTe QDs/nano-Fe_3_O_4_	fluorescent	malachite green	EGDMA	MAA	H_2_O	AIBN	Bulk	8	20–80	14.30	7.04	[256]
CdTe-MPA	fluorescent	histamine	EGDMA	MAA	ethanol	AIBN	Bulk	5	0.01–20	3.70 × 10^−3^	2.397 × 10^−2^ and 0.1966	[257]
CsPbBr_3_/CsPbI_3_@SiO_2_	fluorescent	rhein	TEOS	APTES	n-hexane	NH_3_·H_2_O	Sol–gel	2	1.5 × 10^−2^–0.135	1.90 × 10^−3^	3.80	[258]
TpPa-2 COFs/CsPbBr_3_ QDs	fluorescent	patulin (PAT)	EGDMA	MAA/6-HNA	Methanol	AIBN	Bulk	12	1.3 × 10^−3^–0.13	1.75 × 10^−4^	26.26	[259]

6-HNA: 6-hydroxy-2-naphthoic acid; AIBN: 2,2′-azobisisobutyronitrile; APTES: 3-aminopropyltriethoxysilane; COFs: covalent organic frameworks; EGDMA: ethylene glycol dimethacrylate; LOD: limit of detection; MAA: methacrylic acid; MPA: 3-mercaptopropionic acid; TEOS: tetraethyl orthosilicate.

## Data Availability

The bibliometric data analyzed in this study were retrieved from Scopus and the Web of Science Core Collection. The data supporting the bibliometric analysis are available from the corresponding author upon reasonable request, subject to the terms and conditions of the respective databases.

## Abbreviations

The main abbreviations used throughout this manuscript are listed below:

AIBN	2,2′-Azobisisobutyronitrile
ANI	aniline
APTES	3-aminopropyltriethoxysilane
AuNPs	Gold nanoparticles
CPE	Carbon paste electrode
CQD	Carbon quantum dot
CV	Cyclic voltammetry
DFT	Density functional theory
DMF	Dimethylformamide
DMSO	Dimethyl sulfoxide
DPV	Differential pulse voltammetry
EGDMA	Ethylene glycol dimethacrylate
EIS	Electrochemical impedance spectroscopy
EDOT	3,4-ethylenedioxythiophene
GCE	Glassy carbon electrode
GO	Graphene oxide
GQD	Graphene quantum dot
HPLC	High-performance liquid chromatography
LOD	Limit of detection
LSV	Linear sweep voltammetry
MAA	Methacrylic acid
MC	Mesoporous carbon
MIP	Molecularly imprinted polymer
MD	Molecular dynamics
MIP-RAM	Restricted access molecularly imprinted polymer
MOF	Metal–organic framework
MPS	3-(methacryloxy)propyltrimethoxysilane
MWCNT	Multi-walled carbon nanotube
NIP	Non-imprinted polymer
o-PD	Ortho-phenylenediamine
PANI	Polyaniline
PPy	Polypyrrole
QCM	Quartz crystal microbalance
QD	Quantum dot
RSD	Relative standard deviation
SERS	Surface-enhanced Raman scattering
SPE	Screen-printed electrode
SPME	Solid-phase microextraction
SWV	Square wave voltammetry
TEOS	Tetraethyl orthosilicate
TRIM	Trimethylolpropane triacrylate
